# Combining QTL Mapping and Transcriptomics to Decipher the Genetic Architecture of Phenolic Compounds Metabolism in the Conifer White Spruce

**DOI:** 10.3389/fpls.2021.675108

**Published:** 2021-05-17

**Authors:** Justine Laoué, Claire Depardieu, Sébastien Gérardi, Manuel Lamothe, Claude Bomal, Aïda Azaiez, Marie-Claude Gros-Louis, Jérôme Laroche, Brian Boyle, Almuth Hammerbacher, Nathalie Isabel, Jean Bousquet

**Affiliations:** ^1^Canada Research Chair in Forest Genomics, Centre for Forest Research and Institute for Systems and Integrative Biology, Université Laval, Québec, QC, Canada; ^2^Natural Resources Canada, Canadian Forest Service, Laurentian Forestry Centre, Québec, QC, Canada; ^3^Institute for Systems and Integrative Biology, Université Laval, Québec, QC, Canada; ^4^Department of Zoology, Entomology, Forestry and Agricultural Biotechnology Institute, University of Pretoria, Pretoria, South Africa

**Keywords:** conifers, phenolic compounds, *Picea glauca*, metabolites, QTL, RNA-Seq, co-regulation

## Abstract

Conifer forests worldwide are becoming increasingly vulnerable to the effects of climate change. Although the production of phenolic compounds (PCs) has been shown to be modulated by biotic and abiotic stresses, the genetic basis underlying the variation in their constitutive production level remains poorly documented in conifers. We used QTL mapping and RNA-Seq to explore the complex polygenic network underlying the constitutive production of PCs in a white spruce (*Picea glauca*) full-sib family for 2 years. QTL detection was performed for nine PCs and differentially expressed genes (DEGs) were identified between individuals with high and low PC contents for five PCs exhibiting stable QTLs across time. A total of 17 QTLs were detected for eight metabolites, including one major QTL explaining up to 91.3% of the neolignan-2 variance. The RNA-Seq analysis highlighted 50 DEGs associated with phenylpropanoid biosynthesis, several key transcription factors, and a subset of 137 genes showing opposite expression patterns in individuals with high levels of the flavonoids gallocatechin and taxifolin glucoside. A total of 19 DEGs co-localized with QTLs. Our findings represent a significant step toward resolving the genomic architecture of PC production in spruce and facilitate the functional characterization of genes and transcriptional networks responsible for differences in constitutive production of PCs in conifers.

## Introduction

Empirical evidence indicates that global warming will increase pressure on conifer forests via the intensification of drought events and the introduction of new insects and pathogens (Dale et al., 2001; Sturrock et al., 2011). As long-lived species, trees largely depend on their mechanical and chemical defenses to survive and reproduce (Davis and Shaw, 2001). Trees can protect themselves by producing secondary metabolites such as terpenes and phenolic compounds including flavonoids, monolignols, phenolic acids, stilbenes, and coumarins (Berini et al., 2018). The constitutive production of phenolic compounds (PCs) is usually thought to confer broad resistance to pathogens and abiotic factors in trees (Francheschi et al., 2005; Kessler, 2015). In spruces (*Picea* spp.), PCs are frequently associated with defense responses against pathogenic fungi (Brignolas et al., 1995; Hammerbacher et al., 2011, 2013, 2014) or pest insects (Brignolas et al., 1998; Faccoli and Schlyter, 2007; Delvas et al., 2011; Schiebe et al., 2012; Warren et al., 2015), and a recent study also highlighted their involvement in drought resistance in *Picea abies* (Schiop et al., 2017). Hence, PCs likely play a major role in climate adaptation in conifers. However, the genetic basis underlying their quantitative variation remains poorly understood (Ralph et al., 2006a; Warren et al., 2015), mainly due to the occurrence of large and complex gene families in conifers, combined with a limited knowledge of their functions (Hamberger and Bohlmann, 2006; Prunier et al., 2016).

The regulatory mechanism of phenylpropanoid biosynthesis is complex and involves several families of transcription factors (TFs) that regulate the expression of downstream genes (Yang et al., 2012). Recent advances showed that the PCs pathway in plants is also controlled at different branches by R2R3-MYB repressors (Cavallini et al., 2015; Ma and Constabel, 2019). The regulation of late biosynthetic anthocyanin and proanthocyanin genes is orchestrated by the ternary MBW complex, involving transcription factors from the R2R3-MYB, basic helix–loop–helix (bHLH) and WD40 classes (Xu et al., 2015; Ma et al., 2018). Although the MBW network is evolutionary conserved in plants (Ramsay and Glover, 2005), the considerable expansion of TF families in plant lineages (Feller et al., 2011) could lead to functionally divergent genes between angiosperms and gymnosperms. The complex regulatory system of PCs metabolism, i.e., genes and their networks of interactions, remains largely undeciphered in conifer species. Recent work evidenced protein-protein interactions among different MYB, bHLH and WDR (Nemesio-Gorriz et al., 2017) and NAC gene family (Dalman et al., 2017) in Norway spruce, while other studies identified a variety of gene families (Hamberger and Bohlmann, 2006; Ralph et al., 2006a; Warren et al., 2015), genes (Celedon et al., 2017) and transcription factors (Bomal et al., 2008; Bedon et al., 2010; Deng and Lu, 2017) playing a role in the regulation of PC pathway.

The majority of PC traits are known to be predominantly polygenic (Külheim et al., 2011; Francisco et al., 2016; Ganthaler et al., 2017), hence the need to deploy genome-wide approaches to study their synthesis. Quantitative trait loci (QTL) mapping was used successfully to uncover the genetic architecture of PCs production in crops (Li et al., 2016; Czyczyło-Mysza et al., 2019) and tree species (Verdu et al., 2014; Caseys et al., 2015). QTL analyses have also been successfully conducted in the Pinaceae, using traits relative to growth and phenology (Pelgas et al., 2011; Prunier et al., 2013) and biotic stress resistance (Porth et al., 2012; Lind et al., 2014). However, these analyses suffer from low resolution in conifers because of the low density of genetic linkage maps currently available and because of the considerable time and resources needed to grow, phenotype and genotype progenies. Recently, several studies in angiosperms showed that combining QTL mapping and transcriptome profiling is a robust approach to identify candidate genes underlying complex traits (Xu et al., 2015; Ye et al., 2017; Zhang et al., 2017; Jian et al., 2019). Transcriptome profiling can be used in combination with genetic mapping to narrow down the number of candidate genes identified by QTL and pointing out key genes involved in the mechanisms of interest. Hence, we used a similar approach to identify key genes involved in the PC pathway in white spruce [*Picea glauca* (Moench) Voss], a conifer species with high genome complexity and for which considerable genomic resources such as a draft genome sequences (Birol et al., 2013; Warren et al., 2015), an annotated expressed gene catalog (Rigault et al., 2011), high-throughput genotyping arrays (Pavy et al., 2013), and a high-density genetic linkage map (Pavy et al., 2017) are currently available.

In this study, we first conducted QTL mapping to assess the genomic architecture of the constitutive production of nine candidate PCs in a white spruce full-sib family. Three flavonoids, one neolignan and one stilbenoid, for which we identified QTLs stable across 2 years of measurement, were further analyzed with RNA-Seq in order to identify key genes involved in the regulatory network of PC metabolism. Differentially expressed genes (DEGs) among individuals displaying contrasting phenotypes (high vs. low metabolite content) were identified and compared with genes located in QTLs.

## Materials and Methods

### Workflow

**Figure 1:**
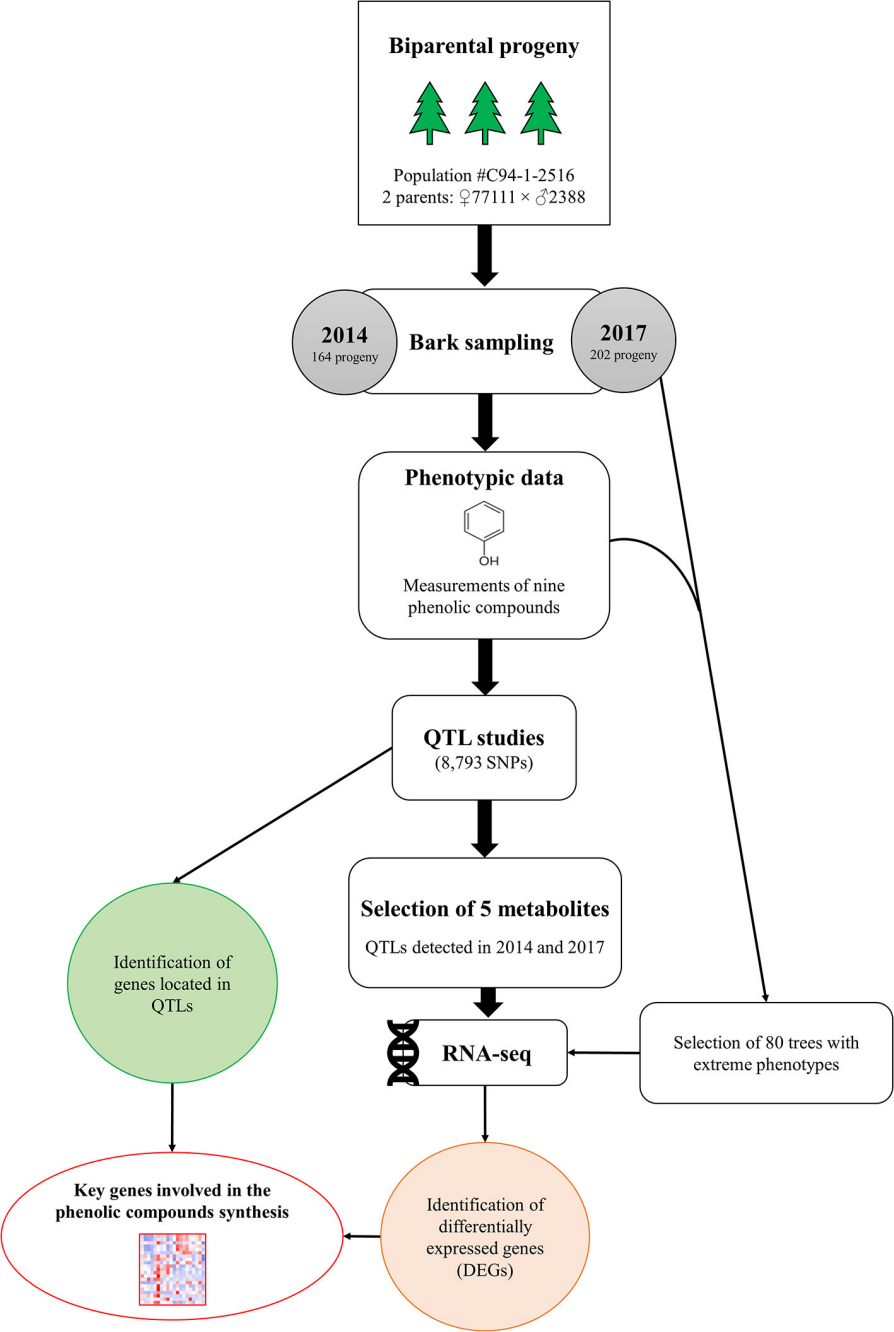
Schematic representation of experimental design. Bark samples were collected from 164 to 202 white spruce siblings in 2014 and 2017, respectively. QTL analyses were performed on nine phenolic compounds for both years. For five metabolites, RNA sequencing was conducted on a subset of individuals displaying high vs. low metabolite contents in 2017. For each of the five metabolites, differentially expressed genes (DEGs) among groups of individuals displaying contrasting phenotypes were identified.

In this study, QTL and RNA-Seq approaches were used to identify genes involved in the metabolism of nine PCs in a random subset of 1,976 siblings previously used to produce a high-resolution genetic map for white spruce (Pavy et al., 2017). The nine PCs, all related to various biotic and abiotic stress responses in spruce species as well as in other plants (Table 1), were quantified for 164 and 202 siblings in two different years (Figure 1). QTL analyses were conducted with phenotypes obtained for the same progeny used for genetic mapping. From there, five metabolites showed a significant underlying genetic component for both years of assessment and were deemed of prime interest to be further investigated at the transcriptomic level using an RNA-Seq approach. For each of these metabolites, at least 20 siblings presenting contrasting phenotypes (i.e., highest and lowest metabolite content) were selected for individual transcriptome profiling and analyzed to highlight sets of differentially expressed genes.

**Table 1:** Bark phenolic compounds analyzed in a white spruce full-sib family.

**Metabolite**	**Molecule skeleton**	**Class**	**MW (g mol^−1^)^a^**	**Formula**	**Biotic stress^b^**	**Abiotic stress^c^**
Astringin	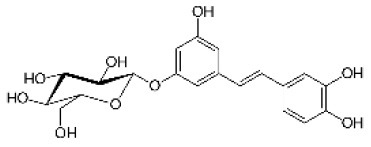	Stilbenoid	406.1	C_20_H_22_O_9_	**Fungus** Hammerbacher et al. (2011)	Not studied
Catechin	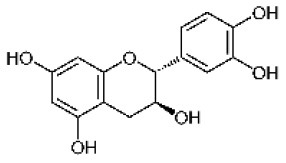	Flavonoid	290.3	C_15_H_14_O_6_	**Fungus** Bahnweg et al. (2000)	Chobot et al. (2009)
Gallocatechin	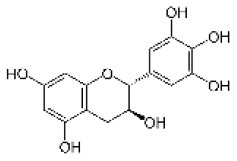	Flavonoid	306.3	C_15_H_14_O_7_	**Fungus** Hammerbacher et al. (2018)	Wang et al. (2016)
Isorhapontin	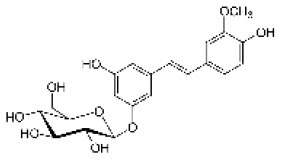	Stilbenoid	420.4	C_21_H_24_O_9_	**Fungus** Hammerbacher et al. (2011)	Not studied
Neolignan-2	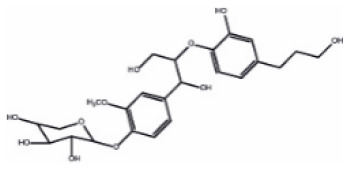	Lignan	400.5	C_23_H_28_O_6_	**Insect** Schiebe et al. (2012)	Moura et al. (2010)
Piceid	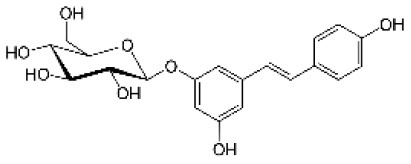	Stilbenoid	390.4	C_20_H_22_O_8_	**Insect and fungus** Brignolas et al. (1998)	Villangó et al. (2016)
Procyanidin B1	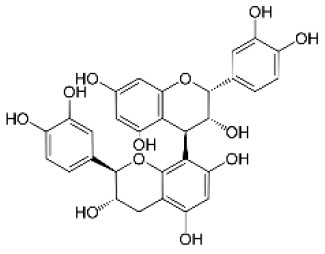	Flavonoid	578.5	C_30_H_26_O_12_	**Insect** Rohde et al. (1996)	Varela et al. (2016)
Taxifolin	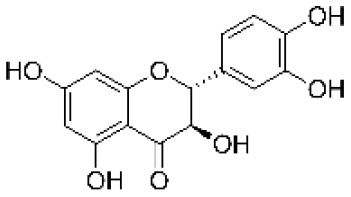	Flavonoid	304.2	C_15_H_12_O_7_	**Fungus** Evensen et al. (2000)	Not studied
Taxifolin glucoside	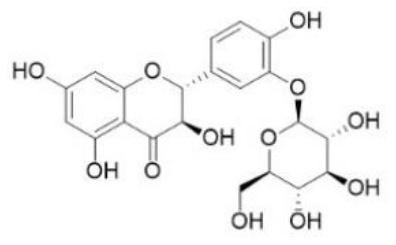	Flavonoid	466.4	C_21_H_22_O_12_	**Insect and fungus** Brignolas et al. (1995)	Not studied

a *Molecular weight of the phenolic compound (g mol^−1^) and chemical formula are indicated*.

b *Studies demonstrating the role of the phenolics tested in tree response to biotic stresses (insect and pathogens) in Picea sp. are reported*.

c *Studies demonstrating the role of the phenolics tested in tree response to abiotic stresses (drought) in plant species are reported*.

### Plant Material and Phenotypic Data Measurements

Twigs were collected on 164 and 202 full-sib siblings in August 2014 (age 15) and August 2017 (age 18), respectively, from a QTL mapping population previously used in several studies (cross C94-1-2516 between ♀77111 × ♂2388; Figure 1; see Pelgas et al., 2011; Pavy et al., 2012, 2017) and raised in a common garden located in Saint-Antonin, Québec (47°45N; 69°28 W). Seventy-nine siblings were common to both sampling years. Twigs (3-4 copies each) corresponding to the two parents originally crossed were also sampled in August 2015, 2016, and 2017 at the Cap-Tourmente Arboretum of Natural Resources Canada, Québec (47°05 N; −70°47 W). All sampled trees were originally produced from grafted material with a normal growth. For each tree, current-year twigs (18 cm long) were collected at breast height on the same side. Each sample was cut longitudinally with a razor blade to separate the woody inner stem tissues, the pith, from the bark tissues. Each sample was then cut into two parts, immediately frozen in liquid nitrogen, and stored at−80°C until further use: one part for metabolite quantification and the other one for transcriptomic analyses.

### Metabolite Quantification: Extraction of Phenolic Compounds

Extraction of PCs, namely astringin, catechin, gallocatechin, isorhapontin, 4-[1,3-dihydroxy-2-[2-hydroxy-4-(3-hydroxypropyl)phenoxy]propyl]-β-D-xylopyranoside (neolignan-2; Supplementary Figure 1), piceid, procyanidin B1, taxifolin and taxifolin glucoside, was performed at the Max Planck Institute in Germany. Briefly, PCs were extracted from ground bark samples and quantified by LC-tandem mass spectrometry on an Agilent 1200 HPLC system (Agilent, Santa Clara, CA, United States) coupled to an API 3200 mass analyzer (Sciex, Darmstadt, Germany). Analyst v1.5 software (Applied Biosystems) was used for data acquisition and processing. Linearity of metabolite detection for quantification was verified by external calibration curves for catechin, taxifolin, astringin and procyanidin B1. Other metabolite concentrations were determined relative to the calibration curve of the metabolite most closely resembling it. Detailed procedures are given in Supplementary Methods 1 in Supplementary Material. Descriptive statistics and phenotypic distributions of metabolites are reported in Supplementary Table 1 and Supplementary Figure 2. Pairwise correlations between metabolite concentrations measured in 2014 and 2017 were calculated with the software R 3.5 (R Core Team, 2013) using the rcor function in the Hmisc R package (Harrell, 2014).

### QTL Analyses

QTL analyses were conducted in order to identify genomic regions accounting for variation in metabolite concentrations among siblings and underlying candidate genes putatively carrying causal variants. QTLs detection was performed for the nine PCs investigated (Table 1). Data were analyzed on a yearly basis using the two parental linkage maps consisting in 2,774 (female) and 2,308 (male) high-quality SNPs mapping in as many separated genetic bins (see details in Pavy et al., 2017), resulting in four distinct QTL analyses per metabolite. All individuals included herein were part of the progeny originally used to generate the consensus linkage map consisting of 8,793 distinct gene loci (Pavy et al., 2017). QTL analyses were conducted with the software MapQTL v6.0 (van Ooijen and Kyazma, 2009), using interval mapping, which is a robust method against deviations from normality (van Ooijen and Kyazma, 2009). A QTL with a LOD score ≥ 3.1 was considered significant (*P* < 0.05 based on 1,000 genome-wide permutations of the markers). QTLs detected on each parental map were then positioned on the consensus map. Genes within 1 LOD of either side of the QTL peak were considered as candidate genes. Given the extremely high LOD score associated with neolignan-2 QTL, the window was expanded to 15 cM, which corresponded to the average window size of other significant QTLs detected in this study.

### RNA-Seq Experiment

**Figure 2:**
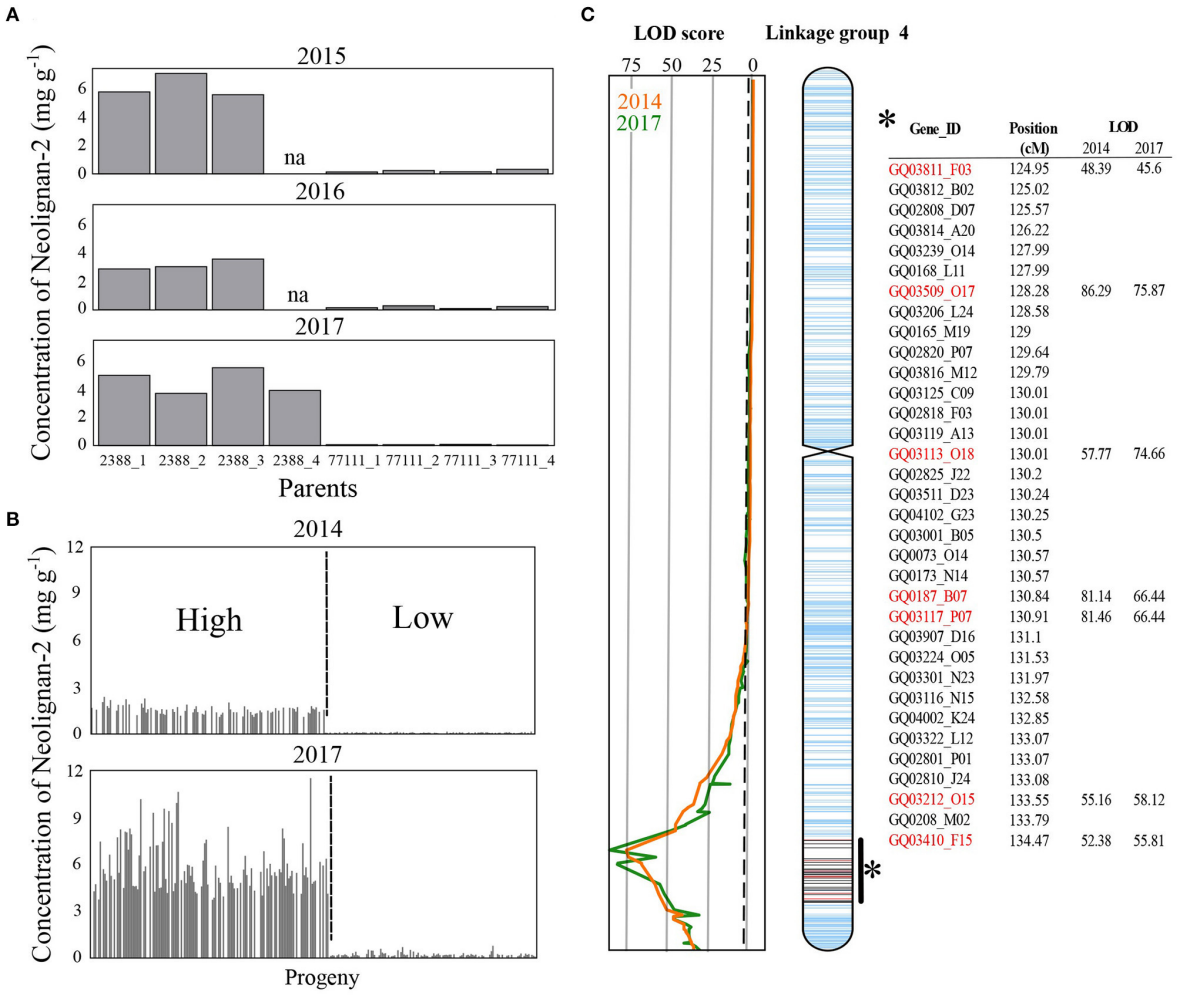
Metabolite concentrations and QTL mapping for neolignan-2. Concentrations of neolignan-2 obtained **(A)** from grafted material (3–4 ramets) corresponding to both parents (♀77111, ♂2388) sampled in August 2015, 2016, and 2017, and **(B)** from 164 and 202 siblings sampled in August 2014 and 2017, respectively. **(C)** Linkage map of the white spruce progeny showing location of the putative QTLs with LOD score for neolignan-2 in 2014 (orange line) and 2017 (green line). The peak LOD score reached 75.87 and 86.29 in 2014 and 2017, respectively. The vertical dotted line indicates the threshold for significant linkages of the LOD scores (i.e., 3.1). Ticks on the chromosome represent mapped genes according to Pavy et al. (2017). Within the QTL interval, red and black ticks are genes for which markers were detected for ♂2388 and ♀77111, respectively.

QTL analyses revealed that five metabolites, namely gallocatechin, neolignan-2, piceid, procyanidin B1 and taxifolin glucoside, had significant QTLs across both years surveyed (Figure 1). The expression of genes involved in the biosynthesis of those five metabolites were further investigated using RNA-Seq to identify sets of differentially expressed genes (DEGs) between two groups of individuals that showed contrasting concentrations of metabolites. Out of the 202 siblings collected in 2017, we selected 80 individuals showing high or low concentrations in gallocatechin, piceid, procyanidin B1 and taxifolin glucoside metabolites (Figure 1), some of them showing extreme phenotypes for more than one metabolite. The transcriptomic profiles of 20 phenotypically divergent individuals per PC (10 high-metabolite content vs. 10 low-metabolite content individuals) were then compared. Since the neolignan-2 displayed a bimodal distribution of concentrations (Figure 2), each of the two contrasted groups of individuals included 35 siblings instead of 10.

#### RNA Extraction, Libraries Preparation and Sequencing

For the 80 individuals selected for RNA-Seq, total RNA was isolated from frozen bark tissues following the method of Chang et al. (1993), with the protocol modifications described in Pavy et al. (2008), as detailed in Supplementary Methods 2 in Supplementary Material. For each sibling, a cDNA library was generated from 1 μg of total RNA using the KAPA stranded mRNA-Seq Kit (KAPA Biosystems, Roche Sequencing solutions, Canada). This kit contains all buffers and enzymes required for the poly(A) mRNA capture and the construction of stranded mRNA-Seq libraries of 100 ng−4 μg of intact total RNA. A PCR of 13 cycles was then performed for each cDNA library having a specific adapter (Illumina TruSeq HT). The quality of total RNA was assessed using the Agilent 2100 Bioanalyzer with RNA 6000 Nano LabChips (Agilent Technologies Inc, Santa Clara, CA, USA) and RNA concentration was determined with a Nanodrop 1000 spectrophotometer (Thermo Fisher Scientific, Wilmington, DE, U.S.A). For all samples, RNA concentration exceeded 100 ng μl^−1^ and RNA Integrity Number (RIN) exceeded 7.8. Libraries were first tagged individually, and then merged into a single sequencing pool at equimolar concentrations. The pool was sequenced at McGill University and Genome Quebec Innovation Center (Montreal, Quebec, Canada) using two lanes of Illumina NovaSeq 6000 S2 PE100.

#### Pre-processing and Differential Gene Expression Analyses

For each of the five metabolites tested, gene expression was compared between groups of individuals showing high and low PC contents. Quality of raw sequence data was first checked using FASTQC v0.11.2 (Andrews et al., 2014) for each sibling sequenced. Adapter sequences were trimmed using Cutadapt (Martin, 2011) and checked again with FASTQC v0.11.2 to ensure proper trimming. For each metabolite, reads of individuals making up the high and low PC content groups were pooled. High-quality reads were then aligned against the most recent version of the white spruce reference transcriptome (Rigault et al., 2011) using Salmon v0.11.0 with the following options: –Gcbias, –seqBias, –validatingMappings and minScoreFraction (0.8) (Patro et al., 2017).

Read counts were further normalized using the DESeq2 Bioconductor package in R (Anders and Huber, 2010; Love et al., 2014). Differential gene expression analyses were then carried out using the default pipeline implemented in the function “DESeq.” The DEGs were then determined using a threshold of < 0.05 (adjusted p-value after applying the Benjamini-Hochberg's multiple-testing correction; Benjamini and Hochberg, 1995). Expression level of DEGs was further classified as high (log_2_fc ≥ 1) or low (log_2_fc ≤ −1) according to the log_2_ fold change values (log_2_fc) obtained when comparing expression in high vs. low metabolite content individuals. DEGs with a total read count lower than 10 were discarded. Venn diagrams were plotted to highlight unique and shared DEGs among metabolites using a bio-analytic web tool (http://bioinformatics.psb.ugent.be/webtools/Venn/).

#### Functional Characterization and Enrichment Tests of Differentially Expressed Genes

Using the BLAST2GO PRO suite (Gotz et al., 2008), functional annotations of the DEGs were retrieved by performing a BLASTX search against the *RefSeq* database (RefSeq release 91, using a cut-off e-value of ≤10^−10^). Homologous protein domains from translated sequences were then identified by searching against the Interpro database. GO annotations (molecular functions and biological processes) were obtained for each DEG and slimmed down into the more general GO plant slim terms. For each metabolite, enrichment tests were performed by comparing GO associated with DEGs with those associated with the full set of expressed genes, using Fisher's exact tests (*P* < 0.05) in the BLAST2GO PRO suite. The transcription factor database PlantRegMap/PlantTFDB v5.0 was used to identify potential transcription factors among DEGs (TFs; http://planttfdb.cbi.pku.edu.cn/; Jin et al., 2017; Tian et al., 2020). Finally, DEG functions were visualized in MapMan v3.5.1 (Thimm et al., 2004) using DEG mapping files generated by Mercator (v3.6; Lohse et al., 2014). MapMan classifies genes into various relevant functional categories, also referred as BIN classes herein.

## Results

### Variation in Phenolic Compounds and Correlations

The nine PCs examined in this study belonged to the flavonoid, the stilbenoid, and the lignan biosynthesis pathways (Table 1). Astringin and isorhapontin were the most abundant metabolites (62.35 and 22.20 mg g^−1^ DW, respectively), followed by taxifolin (12.55 mg g^−1^ DW). The other PCs were relatively less abundant, with concentrations <3.28 mg g^−1^ DW (Supplementary Table 1). All metabolites followed a unimodal and continuous distribution (Supplementary Figure 2), except for neolignan-2, which displayed a clear bimodal distribution in parents (Figure 2A) and progeny (Figure 2B), suggesting the possibility of a major gene effect.

**Figure 3:**
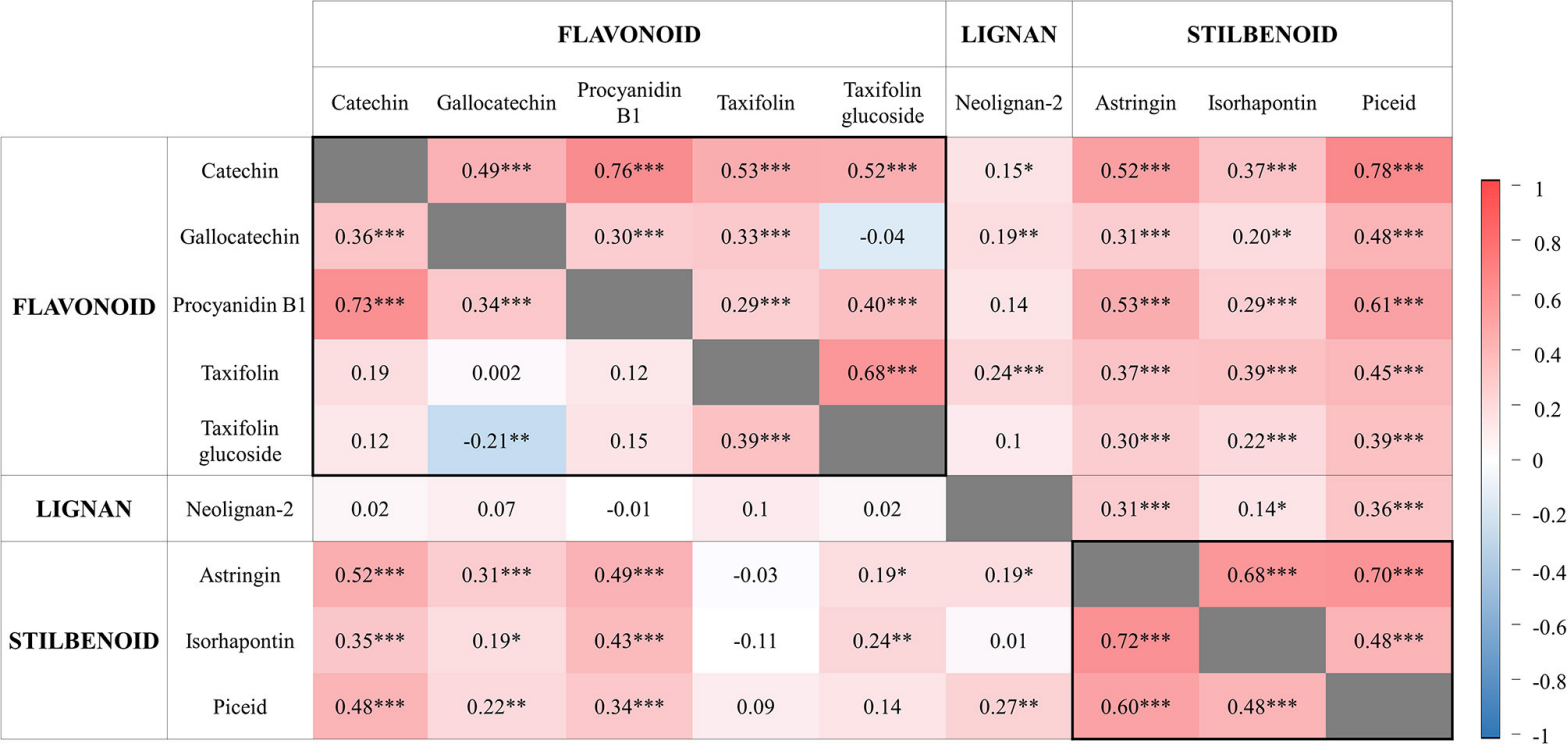
Pairwise correlations between phenolic compounds contents measured in 2014 (bottom left; 164 siblings) and 2017 (top right; 202 siblings). Metabolites are classified into three major classes. The scale bar reports positive (red) and negative (blue) correlations. For each phenotypic correlation, the correlation coefficient and significance level are indicated. Levels of significance are as follows: *P < 0.05, **P < 0.01, and ***P < 0.001.

Most metabolite concentrations within individuals were positively correlated (Figure 3). An exception was found for gallocatechin and taxifolin glucoside, which appeared negatively correlated in 2014 (Figure 3). As a general trend, PCs synthesized from the same biosynthetic pathway were most highly correlated, as observed for the two stilbenoids astringin and piceid (*r* = 0.60 in 2014 and *r* = 0.70 in 2017), and for the flavonoids catechin and procyanidin B1 (*r* > 0.73). However, one other exception was the strong correlation between piceid (a stilbenoid) and catechin (a flavonoid) (*r* > 0.78 in 2017). The concentration of neolignan-2 only moderately correlated with other metabolites (0.14 < *r* < 0.36), but variations in concentration among individuals were stable across time (*r* = 0.94, *P* < 0.05; based on the 79 individuals measured in both years; data not shown).

### QTL Analyses

A total of 17 significant QTLs (LOD score ≥ 3.1) were detected for eight of the nine metabolites tested (Table 2). Individual QTLs included between 23 and 160 genes, resulting in a list of 871 unique candidate genes (Supplementary Table 2). QTLs for astringin, catechin and taxifolin were only detected from 2017 data (Table 2). Significant QTLs for gallocatechin, neolignan-2, piceid, and procyanidin B1 were observed for both years on at least one co-localizing region of a unique parent. In addition, two distinct QTLs were detected for taxifolin glucoside in 2014 and 2017, on different parents and genomic regions. The phenotypic variance explained (PVE) by single QTLs ranged between 8.0% (which is approximately corresponding to the genome-wide significance threshold for this experiment) and 91.3%. Interestingly, very high LOD scores (86.3 and 75.9) and PVE values (91.3 and 82.4%) were obtained for the only QTL detected for neolignan-2 in both years (Table 2; Figure 2C). Those observations highlight the occurrence of a genetic effect with strong impact on this phenotype on linkage group 4 (Figure 2C). Overall, QTLs were located on seven out of the 12 linkage groups (LG), corresponding to the 12 chromosomes of white spruce. Co-localization of QTLs for both years of measurement was observed for four PC (gallocatechin on LG8; neolignan-2 and piceid on LG4; and procyanidin B1 on LG1). This indicates that genotype-phenotype linkages were robust to inter-annual climate variations, which strengthen the involvement of these regions in the control of the studied phenotypes. These compounds were thus selected for transcriptomic analysis along with taxifolin glucoside, for which QTLs were detected for both years of measurement, but on different parents (Table 2; Figure 1).

**Table 2:** Summary statistics of significant QTLs (LOD score ≥ 3.1) detected for eight phenolic compounds in the C94-1-2516 white spruce full-sib family in 2014 and 2017.

**Trait**	**Year**	**Parent**	**Linkage group^a^**	**QTL**				
				**Position^b^**	**LOD score max^c^**	**PVE^d^**	**Number of genes in the QTL^e^**	**Marker with highest LOD^f^**
Astringin	2014			No QTL found				
	2017	77111	2	107.1–124.3	4.33	9.4	101	GQ0201_C16.1:213
Catechin	2014	2388	6	129–137.3	3.31	8.9	74	GQ03417_E16.1:213
								GQ03517_A20.1:183
								GQ03813_N18.1:900
	2017			No QTL found				
Gallocatechin	2014	77111	6	102.6–115.2	4.26	11.3	52	GQ04010_D06.1:89 (LOD 4.21)
		2388	8	80.5–95.6	8.23	20.8	51	GQ03706_F01.1:209 (LOD 8.16)
								PGLM1-0902 (LOD 8.16)
								GQ03222_J15.1:397 (LOD 8.16)
	2017	77111	4	68.1–103.8	4.15	9.1	160	PGLM2-1250
		77111	8	94.1–117.8	3.88	8.5	99	GQ04108_O24.1:246
		2388	8	85.8–99.3	6.93	14.7	52	GQ03616_J03.1:82
Neolignan-2^g^	2014	2388	4	122.3–134.5	86.29	91.3	67	GQ03509_O17.1:634
	2017	2388	4	122.3–134.5	75.87	82.4	61	GQ03509_O17.1:634
Piceid	2014	77111	4	150.3–163.5	7.41	19.1	72	GQ0168_L11.2:1112
	2017	77111	4	151.3–157.2	4.83	10.5	23	GQ02820_P07.1:861
Procyanidin B1	2014	2388	1	0.5–6.2	3.44	9.4	36	GQ03608_I02.1:558 (LOD 3.24)
								GQ03711_A03.1:507 (LOD 3.24)
								PGLM2-0208 (LOD 3.24)
	2017	2388	1	5.2–27.3	4.97	10.8	100	GQ03222_P17.1:624
								GQ03230_C18.1:470
								GQ03718_P22.2:170
Taxifolin	2014			No QTL found				
	2017	77111	10	106.4–124.2	4.22	9.2	60	GQ02811_E24.1:907
								GQ03001_G19.1:192
								GQ02802_M06.1:581
								PGLM1-0106
								GQ0024_A06.1:139
			6	102.6–111.1	3.66	8.0	35	WS00110_I05.1:387 (LOD 3.63)
Taxifolin glucoside	2014	2388	6	63.5–72.3	4.18	11.7	38	GQ03220_G12.1:1305 (LOD 4.01)
	2017	77111	9	0.0–8.9	3.81	8.4	27	PGLM2-0975 (LOD 3.79)
								GQ03613_M22.1:156 (LOD 3.79)
								PGLM1-1021 (LOD 3.79)

a *Linkage group (LG) according to Pavy et al. (2017)*.

b *Position on the consensus map ± 1 LOD in centimorgan (cM)*.

c *LOD score max: maximum LOD score for mapped markers*.

d *PVE: phenotypic variance explained, expressed in percentage (%)*.

e *Number of genes found in the QTL*.

f *Associated marker (Pavy et al., 2017) with highest LOD. If no marker was present at the highest LOD score, the marker closest to the LOD score max was indicated and its LOD score in parenthesis*.

g *QTL position for neolignan-2 was defined as the average window size of significant QTLs detected in other metabolites (e.g., 15 cM), as only one gene was mapped in the neolignan-2 QTL when using a window of ± 1LOD*.

The functional annotation of genes located within QTL regions relied on BLAST2GO annotations, GO terms classification, and protein family signatures. While most QTL genes had no known direct role in the phenylpropanoid pathway, we found two genes previously reported as involved in the PC biosynthesis in *Picea abies*, namely a leucoanthocyanidin reductase-like gene (LAR; GQ03701_M12) for procyanidin B1, and a flavonoid 3′,5′-hydroxylase 2-like gene (F3′5′H; GQ03712_G11) for taxifolin glucoside (Supplementary Table 2).

### Relative Functions of Differentially Expressed Genes Between Groups of High and Low PC Content Individuals

**Figure 4:**
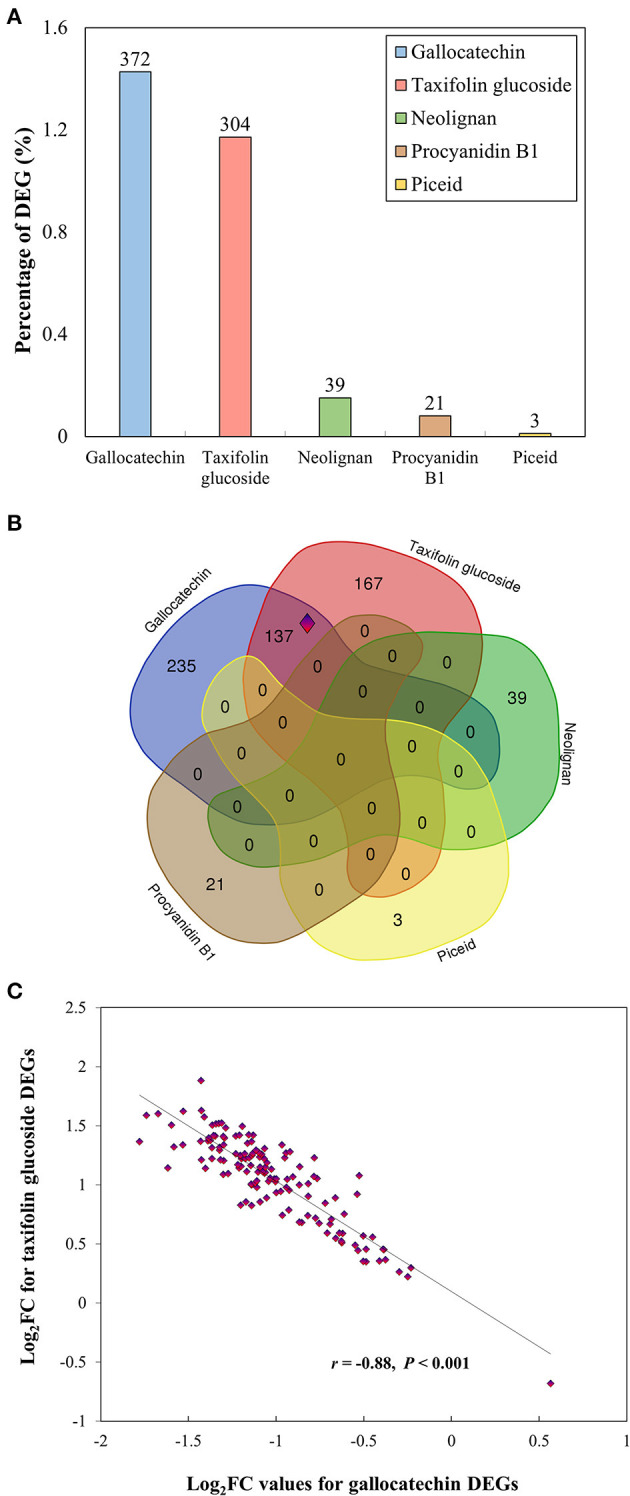
Results of differential expression analyses for five phenolic compounds. **(A)** Proportion of differentially expressed genes (DEGs) among groups of individuals displaying contrasting phenotypes, for each metabolite. The proportion of DEGs was calculated as the number of DEGs divided by the total number of genes expressed. The number of DEGs is reported above the bars. **(B)** Venn diagrams showing the overlap among the 603 DEGs identified for all five metabolites. The set of 137 genes shared between gallocatechin and taxifolin glucoside (surrounded by a blue and red diamond) is further presented in panel (c). **(C)** Opposite expression profiles of the 137 genes shared between gallocatechin and taxifolin glucoside. The log fold changes of DEGs identified for taxifolin glucoside are plotted against log fold changes of DEGs identified for gallocatechin. The correlation coefficient and *p*-values are reported. Log_2_FC: log_2_ fold change.

The five selected metabolites (gallocatechin, neolignan-2, piceid, procyanidin B1, and taxifolin glucoside) were further investigated using RNA-Seq to compare the transcriptomic profiles of individuals showing contrasting phenotypes (high vs. low metabolite concentrations for each PC). A summary of RNA-Seq statistics is presented in Supplementary Table 3. A total of 603 unique DEGs were identified at a rate of 3 to 372 DEGs per metabolite (Figure 4A; Supplementary Table 4). DEGs overlap among metabolites was very limited to non-existent, except for gallocatechin and taxifolin glucoside, which shared 137 DEGs (Figure 4B). Except for one gene, these DEGs showed higher expression in high taxifolin glucoside content individuals and lower expression in high gallocatechin content individuals (Figure 4C). Among the 603 DEGs, 436 were successfully annotated with a GO term associated to molecular functions and biological processes (Supplementary Figure 3). The GO enrichment analyses revealed that DEGs were particularly overrepresented in monosaccharide metabolic (2.5%), secondary metabolic (1.9%) and phenylpropanoid metabolic (1.9%) processes (Supplementary Figure 3). Overrepresented molecular functions included stress-related functions such as catalytic (59.2%), oxidoreductase (16.8%), lyase (7.6%), and transferase (5.7%) activities, and functions related to the phenylpropanoid pathway such as UDP-glycosyltransferase and O-methyltransferase activities (Supplementary Figure 3).

**Figure 5:**
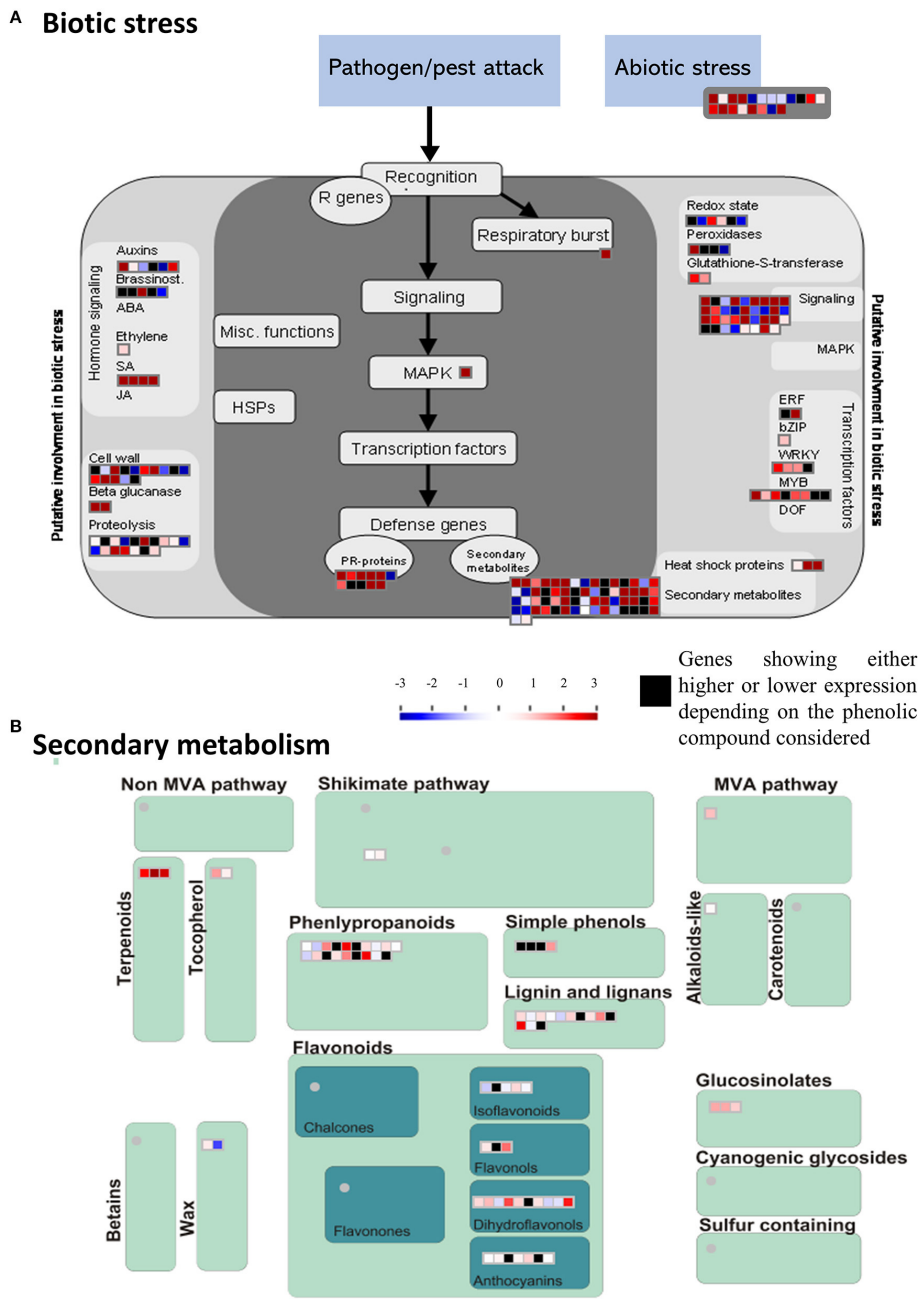
MapMan-based classification of differentially expressed genes (DEGs) involved in stress responses. **(A)** Expression profiles of DEGs involved in biotic and abiotic stresses. **(B)** Expression profiles of DEGs involved in secondary metabolism. The scale bar represents positive (red) and negative (blue) regulation of gene expression based on log_2_fc scores. Black squares represent genes that were either high or low expressed depending on the phenolic compound considered.

MapMan analyses were conducted to classify DEGs into relevant functional bins (or functional categories; Supplementary Figure 4; Supplementary Table 5). DEGs were assigned to 29 functional bins. While an important proportion of the 603 DEGs were not assigned (37%), a significant proportion of DEGs was related to secondary metabolism (8.9%), stress (5.9%) and signaling (5%) (Supplementary Figure 4). The stress-related genes included 20 unique DEGs involved in abiotic stress response (Figure 5A). The most relevant functions associated to the 144 unique DEGs involved in biotic stress response included pathogenesis-related (PR) proteins, heat shock proteins, transcription factors as well as various genes involved in hormone signaling and defense (Figure 5A). The 59 defense-related DEGs identified by MapMan (Figure 5A) included 11 putative PR-proteins, and 48 genes involved in secondary metabolism, among which 36 genes were involved in the shikimate/phenylpropanoid pathway (Figure 5B).

### Identification of Candidate Genes Specifically Associated to the Phenylpropanoid Pathway

For all metabolites considered, the combination of MapMan classification and Blast2GO functional annotations allowed for the identification of 50 DEGs involved in the phenylpropanoid pathway associated with monophenols, phenylpropanoids, flavonoids, as well as lignins and lignans metabolism (Table 3; Figure 5B). From this subset, 31 and 30 DEGs were respectively associated to gallocatechin and taxifolin glucoside, including 15 genes shared between the two DEG sets. In addition, four other genes were associated with neolignan-2 and procyanidin B1. For all metabolites, the most represented gene families were the putative dirigent proteins (DIR) (9 genes), laccases (7 genes) and plant peroxidases (6 genes) (Table 3). Among the 50 DEGs identified, 36 DEGs were differentially expressed between the groups of high and low PC content individuals. A large proportion of putative DIR showed higher expression, with three genes associated with taxifolin glucoside (GQ03808_J11, GQ03806_D05, GQ03307_E08), two with gallocatechin (GQ036606_J07, GQ03815_M16), one with neolignan-2 (WS00740_J05) and one with procyanidin B1 (GQ01301_K10). Two DIR showing lower expression were detected for gallocatechin (GQ03806_D05, GQ03803_O03). Most laccases associated to the phenylpropanoid pathway were also differentially expressed, with four highly expressed and three lower expressed genes in individuals with high taxifolin glucoside and high gallocatechin content, respectively. In addition, three putative peroxidases (GQ0202_L09, GQ03322_C02, GQ02016_E21), two O-methyltransferases (WS00740_E09, GQ03507_F11), one cytochrome P450 (WS00736_D10) and one NmrA-like (GQ03009_B07) putative protein showed higher expression in high taxifolin glucoside content individuals.

**Table 3:** List of differentially expressed genes (DEGs; adjusted *P* < 0.05) involved in phenolic compounds metabolism.

**Metabolite**	**DEG ID**	**Sequence description^a^**	**InterPro classification^b^**	**Adjusted *p*-values**	**Log_**2**_fc**	**Expression^c^**
Gallocatechin	GQ04107_C21	Flavonoid 3′,5′-hydroxylase 2-like	Cytochrome P450 E-class group I	4.14E-05	2.30	HIGH
	GQ03606_J07	Dirigent protein 11-like	Dirigent protein	4.41E-02	2.20	HIGH
	GQ04105_L05	Protein DMR6-LIKE OXYGENASE 2	Isopenicillin N synthase-like, oxoglutarate/iron-dependent dioxygenase	6.39E-03	2.05	HIGH
	GQ03815_M16	Dirigent protein 1-like	Dirigent protein	1.82E-03	1.76	HIGH
	GQ03111_E17	Probable mannitol dehydrogenase	Leucine-rich repeat domain superfamily	7.62E-03	−1.17	LOW
	GQ03313_A02	Cinnamoyl-coa reductase 1-like	NAD-dependent epimerase/dehydratase	2.65E-02	−1.00	LOW
	GQ03805_H10	Laccase-3-like isoform X1	Cupredoxin, laccase, multicopper oxidase type 1	9.81E-03	−1.07	LOW
	GQ03806_D05	Dirigent protein 11	Dirigent protein	1.72E-02	−1.08	LOW
	GQ02901_F15	Bifunctional pinoresinol-lariciresinol reductase 2	NmrA-like domain	3.38E-03	−1.10	LOW
	GQ03322_C02	Peroxidase 11	Plant peroxidase	3.04E-02	−1.11	LOW
	GQ03216_M13	Laccase-5-like	Laccase, multicopper oxidase type 1	7.00E-03	−1.15	LOW
	GQ03812_J09	Xanthohumol 4'-O-methyltransferase	Winged helix-like DNA-binding domain superfamily, O-methyltransferase domain	4.56E-03	−1.17	LOW
	GQ03803_O03	Dirigent protein 22-like	Dirigent protein	2.07E-03	−1.20	LOW
	GQ0202_L09	Peroxidase 72-like	Plant peroxidase	2.45E-03	−1.22	LOW
	GQ03009_B07	Isoflavone reductase homolog PCBER-like	NmrA-like domain	2.61E-04	−1.30	LOW
	GQ03807_A11	Omega-hydroxypalmitate O-feruloyl transferase	Chloramphenicol acetyltransferase-like domain superfamily	7.05E-03	−1.31	LOW
	GQ03214_N14	Laccase-12-like	Cupredoxin, multicopper oxidase type 2	4.07E-03	−1.39	LOW
	GQ0253_H12	UDP-glycosyltransferase 85A8-like	FAD/NAD(P)-binding domain superfamily	1.18E-03	−1.43	LOW
	GQ03804_M07	Peroxidase 40	Plant peroxidase	1.44E-02	−1.58	LOW
	GQ0082_B18	Flavonol synthase/flavanone 3-hydroxylase-like	Isopenicillin N synthase-like	8.28E-06	−1.74	LOW
	GQ03229_E14	UDP-glycosyltransferase 86A1	Alpha/Beta hydrolase fold	6.39E-03	1.00	−
	GQ03507_H08	Isoflavone reductase homolog TP7	Concanavalin A-like lectin/glucanase domain superfamily	3.50E-02	0.94	−
	GQ03901_P05	Probable 2-oxoglutarate-dependent dioxygenase ANS	Isopenicillin N synthase-like	2.39E-02	0.53	−
	GQ03810_P08	Isoflavone reductase-like protein	NmrA-like domain	3.77E-03	−0.47	−
	GQ03312_B13	Phenylalanine ammonia-lyase	Phenylalanine ammonia-lyase shielding domain superfamily	2.11E-02	−0.56	−
	GQ0043_N14	Anthranilate N-methyltransferase-like	O-methyltransferase domain	1.02E-03	−0.58	−
	GQ03207_H04	Isoflavone reductase homolog A622-like	NmrA-like domain	2.49E-03	−0.60	−
	GQ03712_G11	Flavonoid 3′,5′-hydroxylase 2-like	Cytochrome P450	3.24E-02	−0.85	−
	WS00736_D10	Cinnamoyl-coa reductase 1-like isoform X2	Cytochrome P450 superfamily	3.06E-02	−0.86	−
	GQ03712_H19	Anthocyanidin reductase ((2S)-flavan-3-ol-forming)	Citrate synthase superfamily	3.50E-04	−0.87	−
	GQ04102_M17	Protein DMR6-LIKE OXYGENASE 2-like	Oxoglutarate/iron-dependent dioxygenase	4.97E-02	−0.91	−
Neolignan-2	WS00740_J05	Dirigent protein 11-like	Dirigent protein	1.01E-05	1.60	HIGH
	GQ03232_H18	Protein DMR6-LIKE OXYGENASE 2-like	Oxoglutarate/iron-dependent dioxygenase	4.25E-04	1.26	HIGH
	GQ02820_P07	Anthranilate N-benzoyltransferase protein 1	Chloramphenicol acetyltransferase-like domain superfamily	6.28E-12	−0.27	−
Procyanidin B1	GQ01301_K10	Disease resistance response protein 206 isoform X2	Dirigent protein	2.11E-02	2.45	HIGH
Taxifolin glucoside	WS00740_E09	Caffeic acid 3-O-methyltransferase	O-methyltransferase domain	6.39E-03	2.51	HIGH
	GQ0253_H12	UDP-glycosyltransferase 85A8-like	FAD/NAD(P)-binding domain superfamily	2.29E-04	1.88	HIGH
	GQ03519_N09	Flavonol synthase/flavanone 3-hydroxylase	Protein kinase-like domain superfamily	7.88E-03	1.82	HIGH
	GQ02016_E21	Peroxidase 72-like isoform X2	Plant peroxidase	1.11E-06	1.74	HIGH
	GQ03507_F11	Caffeic acid 3-O-methyltransferase-like	O-methyltransferase COMT-type	2.92E-02	1.67	HIGH
	GQ0082_B18	Flavonol synthase/flavanone 3-hydroxylase-like	Isopenicillin N synthase-like	1.10E-04	1.59	HIGH
	GQ03805_O13	Laccase-3-like	Multicopper oxidase type 2	5.88E-04	1.53	HIGH
	GQ03807_A11	Omega-hydroxypalmitate O-feruloyl transferase	Chloramphenicol acetyltransferase-like domain superfamily	6.55E-08	1.52	HIGH
	GQ03808_J11	Dirigent protein 22-like	Dirigent protein	8.72E-04	1.41	HIGH
	GQ03214_N14	Laccase-12-like	Cupredoxin, multicopper oxidase type 2	3.42E-04	1.37	HIGH
	GQ03805_H10	Laccase-3-like isoform X1	Cupredoxin, laccase, multicopper oxidase type 1	4.69E-04	1.31	HIGH
	GQ03806_D05	Dirigent protein 11	Dirigent protein	4.17E-04	1.26	HIGH
	GQ03216_M13	Laccase-5-like	Laccase, multicopper oxidase type 1	9.87E-05	1.23	HIGH
	GQ03812_J09	Xanthohumol 4'-O-methyltransferase	Winged helix-like DNA-binding domain superfamily	1.35E-05	1.22	HIGH
	GQ03307_E08	Disease resistance response protein 206 precursor	Dirigent protein	3.04E-05	1.22	HIGH
	GQ0202_L09	Peroxidase 72-like	Plant peroxidase	4.20E-04	1.17	HIGH
	WS00736_D10	Cinnamoyl-coa reductase 1-like isoform X2	Cytochrome P450 superfamily	2.33E-02	1.15	HIGH
	GQ03111_E17	Probable mannitol dehydrogenase	Leucine-rich repeat domain superfamily	3.26E-03	1.11	HIGH
	GQ03009_B07	Isoflavone reductase homolog PCBER-like	NmrA-like domain	9.14E-04	1.09	HIGH
	GQ03322_C02	Peroxidase 11	Plant peroxidase	9.84E-03	1.04	HIGH
	GQ03814_I06	Cinnamoyl-coa reductase 1-like	NAD-dependent epimerase/dehydratase	3.70E-02	1.03	HIGH
	GQ03206_H08	Dihydroflavonol 4-reductase-like	NAD-dependent epimerase/dehydratase	1.06E-02	1.03	HIGH
	GQ04004_H10	Geraniol 8-hydroxylase-like	Cytochrome P450 superfamily	2.93E-02	−1.00	LOW
	GQ03712_H19	Anthocyanidin reductase ((2S)-flavan-3-ol-forming)	NAD-dependent epimerase/dehydratase	1.50E-04	1.00	−
	GQ0074_I15	Hydroquinone glucosyltransferase-like	LysM domain	1.91E-04	0.93	−
	GQ03004_G22	Phenylalanine ammonia-lyase	Phenylalanine ammonia-lyase shielding domain superfamily	1.89E-02	0.92	−
	WS0322_G20	4-coumarate–coa ligase 2	B-cell receptor-associated protein 29/31	1.70E-03	0.87	−
	GQ03321_M15	Cytochrome P450 CYP736A12-like	Ribosomal protein S11, cytochrome P450 E-class group I superfamily	2.06E-02	0.83	−
	GQ03803_O03	Dirigent protein 22-like	Dirigent protein	3.18E-02	0.83	−
	GQ03313_I03	Protein SRG1	Isopenicillin N synthase-like, Oxoglutarate	2.57E-02	0.75	−

a *Sequence description: annotations obtained using BLAST2GO (P < 0.05)*.

b *InterPro classification: most informative InterPro names*.

c *Expression: DEGs having higher expression (log2 fold change ≥ 1) in trees producing high levels of PCs were labeled as HIGH, while DEGs having lower expression (log2 fold change ≤ −1) in individuals producing high levels of PCs were labeled as LOW. Piceid does not appear in the table as none of the 3 DEGs identified for this metabolite was associated with the phenylpropanoid pathway*.

**Figure 6:**
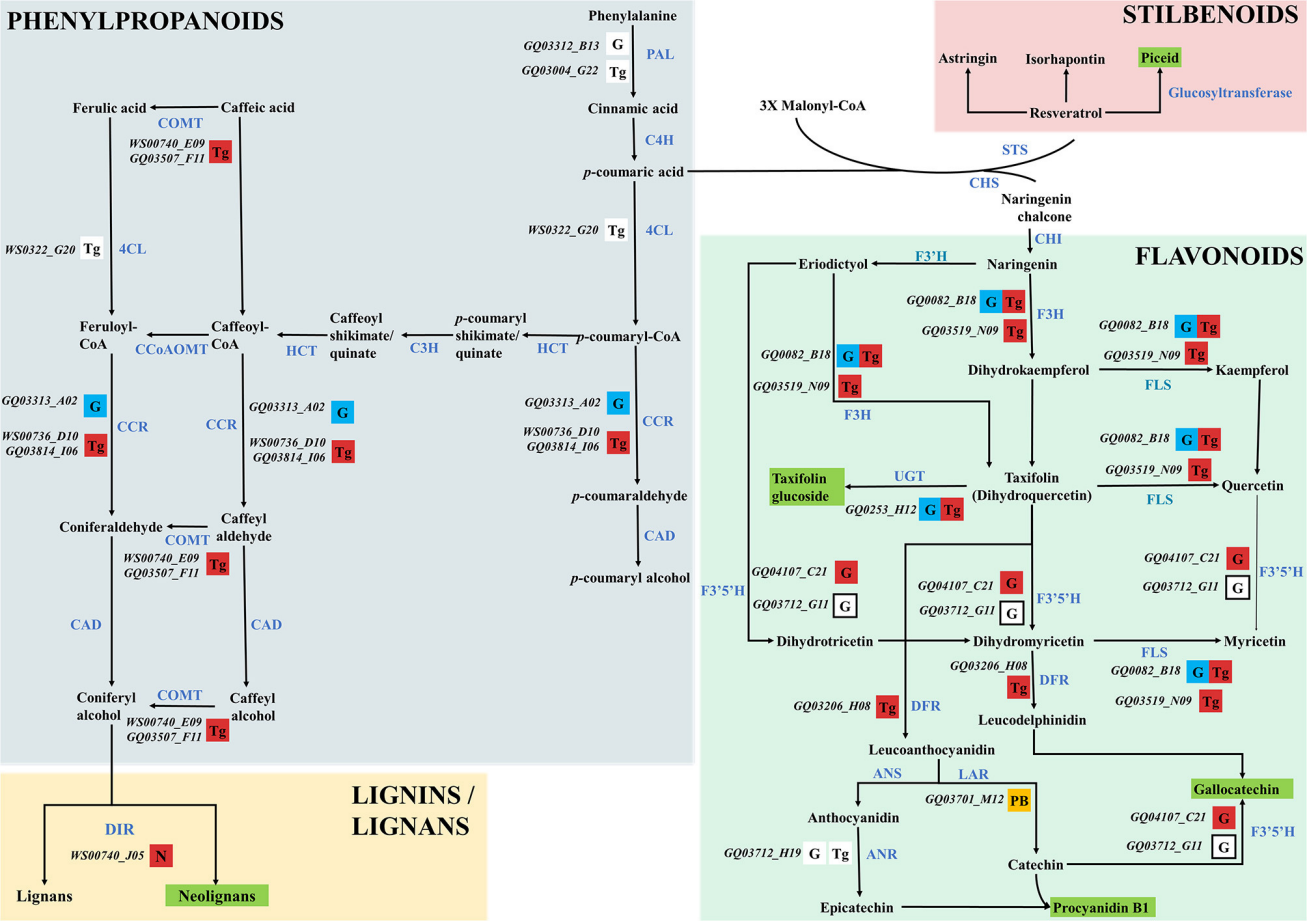
Proposed phenolic compound biosynthesis pathway in spruce. The pathway presented here is adapted from Warren et al. (2015). Candidate gene IDs identified in this study were retrieved from the gene catalog GCAT3.3 (Rigault et al., 2011) and are reported in black and italic. Genes identified in QTLs only are shown in orange boxes. Lower- or highly-expressed genes are indicated by blue and red boxes, respectively. Remaining DEGs (i.e., excluding high- and low expressed genes) are shown in white boxes. Genes within the taxifolin glucoside QTL and differentially expressed for gallocatechin are shown in white boxes with bold margins. The metabolite for which genes are differentially expressed is reported in each box according to the following nomenclature: G, gallocatechin; N, neolignan-2; P, piceid; PB, procyanidin B1; Tg, taxifolin glucoside. The five metabolites studied are underlined in green. Known key enzymes involved in the phenylpropanoid pathway are labeled in blue and bold capital letters. Abbreviations are as follow: 4CL, 4-coumarate-CoA ligase; ANR, anthocyanidin reductase; ANS, anthocyanidin synthase; C3H, p-coumaroyl shikimate/quinate 3′ -hydroxylase; C4H, cinnamate 4-hydroxylase; CAD, cinnamyl-alcohol dehydrogenase; CAD, cinamyl alcohol deshydrogenase; CCoAOMT, caffeoyl-CoA O-methyltransferase; CCR, cinnamoyl-CoA reductase; CHI, chalcone isomerase; CHS, naringenin-chalcone synthase; COMT, caffeic O-methyltransferase DFR, bifunctional dihydroflavonol 4-reductase/flavanone 4-reductase; DIR, dirigent protein; F3H, flavanone 3-hydroxylase; F3′H, flavonoid 3′-hydroxylase; F3′5′H, flavonoid 3′5′-hydroxylase; FLS, flavonol synthase; HCT, hydroxycinnamoyl-CoA:shikimate/quinate hydroxycinnamoyltransferase; LAC, laccase; LAR, leucoanthocyanidin reductase; PAL, phenylalanine ammonia-lyase; PER, peroxidase; PLR, pinoresinol lariciresinol reductase; STS, trihydroxystilbene synthase; UGT, UDP-dependent glucosyl transferase.

In this study, genes known to be involved in the phenylpropanoid pathway in white spruce or other plant species, and genes that were found differentially expressed were designated as key genes. Among the 50 DEGs associated to the phenylpropanoid pathway (Table 3), we identified five key DEGs, namely the 4-coumarate-CoA ligase (4CL), the phenylalanine ammonia-lyase (PAL), cinnamoyl-CoA reductase (CCR), *p*-coumaroyl shikimate/quinate 3′–hydroxylase (C3H) and caffeic *O*-methyltransferase (COMT) (Figure 6). Six other key enzymes were associated specifically to the flavonoid pathway, and included the flavonol synthase (FLS), the UDP-dependent glucosyl transferase (UGT), the flavonoid 3′5′-hydroxylase (F3′5′H - CYP75A), the bifunctional dihydroflavonol 4-reductase/flavanone 4-reductase (DFR), the leucoanthocyanidin reductase (LAR) and the anthocyanidin reductase (ANR) (Figure 6). It should be noted that FLS, UGT and F3H displayed opposite gene expression patterns in individuals producing high levels of taxifolin glucoside, compared to those producing high levels of gallocatechin (see Supplementary Table 4 for details).

### Differentially Expressed Transcription Factors as Potential Regulators of the Phenylpropanoid Pathway

A total of 41 transcription factors (TFs) were differentially expressed among the high and low PCs content groups (Supplementary Table 6). Among those, MYB (10 genes), WRKY (5 genes), NAC and AP2-EREBP (3 genes) were the most represented families. No significant TFs were found for piceid, one for procyanidin B1, while only three TFs were detected for neolignan-2. The remaining TFs were associated to taxifolin glucoside and gallocatechin. One bHLH (GQ01301_E17), one WD40 (GQ03304_I14), one WRKY (GQ03304_E19) and eight MYBs genes showed high and low expression in individuals producing high levels of taxifolin glucoside and gallocatechin, respectively.

### Combining QTL and Transcriptomic Analyses to Identify Candidate Genes for Phenolic Compound Variation

A total of 19 DEGs that were associated to gallocatechin, neolignan-2, and taxifolin glucoside, were also located within QTL regions (Supplementary Table 7). Those genes can be safely designated as candidate genes for PC variation. They included five hydrolases, four uncharacterized putative proteins as well as two genes encoding methylesterases and transferases. None of them were formally associated to the phenylpropanoid pathway.

## Discussion

### Neolignan: A Likely Rare Case of Monogenic Trait in Conifers

Two types of phenotypic distributions were observed in the present study (Figure 2; Supplementary Figure 2). Stilbenoids and flavonoids displayed a continuous and unimodal distribution of concentrations typical for quantitative traits controlled by multiple genes with small effects (Supplementary Figure 2), as generally observed for Pinaceae taxa (Routaboul et al., 2012; Wahyuni et al., 2014; Ganthaler et al., 2017). The distribution of their 15 QTLs across 7 linkage groups (Table 2), the moderate proportion of variance explained by single QTLs (10.9% ≤ PVE ≤ 13%; Table 2), and the distribution of 197 DEGs across the 12 spruce linkage groups (Supplementary Table 2) further support the scenario of a polygenic control of stilbenoid and flavonoid production. In contrast, neolignan-2 displayed a bimodal distribution of metabolite content (Figure 2). This distribution suggests a monogenic control of metabolite concentration, consistent with the identification of a single major QTL on LG4 (Figure 2C) explaining up to 91.3% of the phenotypic variance (Table 2). Until now, studies reporting the occurrence of neolignans in the *Picea* genus, and more generally in conifers, are still scarce [but see Hong et al. (2014); for review see Tanase et al. (2019)]. The neolignan analyzed in the current study (neolignan-2) was recently identified in Norway spruce (Nemesio-Gorriz et al., 2017), and found under the regulation of several MYB genes. Neolignans have diverse physiological roles in angiosperm plants, including defense against fungus and insects (Choi et al., 2009; Saguez et al., 2013), but little is known regarding their specific role in conifers. Monogenic traits usually represent prime candidates for marker-assisted selection in breeding programs. In this sense, further research should focus on the characterization and physiological role of neolignan-2 in white spruce, and densifying the QTL involved with more gene markers, given that less than one third of the transcriptome has been mapped in white spruce (Pavy et al., 2017).

### Metabolite Data and Expression Profiles Reveal New Insights Into the Transcriptional Control of Flavonoids

Transcriptomic data highlighted a set of 137 genes showing opposite expression patterns between high gallocatechin and high taxifolin glucoside individuals (*r* = −0.88, *P* < 0.001; Figure 4). While further investigations are needed to determine which of these genes play a role in the constitutive production of these two flavonoids, such a strong negative relationship suggests that these 137 genes are likely co-regulated, as commonly observed for genes related to the biosynthesis of flavonoids (Honda et al., 2002; Tian et al., 2017). This opposite expression pattern also points to a possible feedback mechanism influencing the production of these two metabolites. However, the lack of significant negative correlation between gallocatechin and taxifolin glucoside concentrations in contrasted phenotypes (Supplementary Figure 5) indicates that gene expression and metabolite levels are decoupled. This does not rule out the possibility of a feedback mechanism, given that metabolite concentrations can be affected by metabolic network connectivity, when metabolites are involved in several metabolic pathways (Wegner and Kummer, 2005; Zelezniak et al., 2014). Alternatively, the activation of these genes, or a subset of them, may simply enhance the production of taxifolin glucoside, without affecting the production of gallocatechin, while their repression would yield an opposite pattern. Since both pathways require the same substrate, this scenario would imply that substrate availability is not a limiting factor for the synthesis of these two metabolites.

### Trancriptomics Highlighted Key Genes Involved in the Phenylpropanoid Pathway and Environmental Stress Response

Transcriptomics revealed that members of gene families generally associated with the phenylpropanoid pathway in plants (Reinprecht et al., 2017) such as DIR, PER-LAC, cytochrome P450, NmrA-like and O-methyltransferases were differentially expressed in white spruce (Table 3; Figure 6). Some key genes involved in the flavonoid pathway (FLS, UGT and F3H; Figure 6) displayed opposite gene expression patterns in individuals producing high levels of taxifolin glucoside and gallocatechin. This observation indicates that these genes are important actors of the transcriptional machinery governing the production of flavonoids. Two genes showing higher expression in trees producing high taxifolin glucoside and gallocatechin contents (GQ03519_N09 and GQ0082_B18; Figure 6) encoded the flavanone 3-hydroxylase (F3H), an enzyme mediating the production of taxifolin in spruce (Hammerbacher et al., 2019). A previous study reported a negative feedback regulation of F3H expression by catechins in *Camellia sinensis* (Singh et al., 2008). Similar mechanism may occur in white spruce, since catechin and taxifolin metabolite levels were strongly positively correlated in trees producing high or low levels of gallocatechin and taxifolin glucoside (Supplementary Figure 5). Expression level of UGT85A8 was higher in individuals producing high levels of taxifolin glucoside, in line with the commonly accepted hypothetical biosynthesis pathway in spruce where UGT facilitated the glucosylation of taxifolin to taxifolin glucoside (Hammerbacher et al., 2019). One gene encoding a flavonoid 3′5′-hydroxylase (F3′5′H; GQ04107_C21) had a higher expression level in high gallocatechin content individuals (Table 3; Figure 6). F3′5′H is an important branch point enzyme in flavonoid biosynthesis that catalyzes the conversion of flavonols into 3′,4′,5′-hydroxylated derivatives and allows the formation of gallocatechin (Deng and Lu, 2017; Figure 6). In Norway spruce, the formation of gallocatechin preferentially leads through catechin rather than dihydromyricetin and leucodelphinidin, and was induced following bark beetle-fungus infection (Hammerbacher et al., 2018, 2019). Finally, one gene located in the procyanidin B1 QTL (GQ03701_M12) encoded the leucoanthocyanidin reductase (LAR) (Figure 6), a bifunctional enzyme catalyzing the reduction of leucocyanidin and controlling the degree of polymerization of proanthocyanidins (Jun et al., 2018; Yu et al., 2019). In Norway spruce, the homologous gene of GQ03701_M12 (Hammerbacher et al., 2014) and other LAR genes (Oliva et al., 2015; Nemesio-Gorriz et al., 2016) were found to be involved in response to fungus infection. Interestingly, one dirigent protein (DIR, WS00740_J05) was highly expressed in individuals with high neolignan-2 content (Table 3; Figure 6). Dirigent proteins are involved in the stereoselective reaction forming the lignan pinoresinol from coniferyl alcohol (Davin and Lewis, 2005), and could also act in the formation of lignin (Burlat et al., 2001). These proteins are usually induced by wounding as well as weevil and budworm herbivory attacks (Ralph et al., 2006b; Lippert et al., 2007).

Phenylpropanoid biosynthesis is a common plant response to biotic and abiotic stress. In line with this idea, several DEGs identified herein were previously found involved in climate adaptation (Supplementary Table 8; Hornoy et al., 2015), cold hardening and cold acclimation in various spruce species (Supplementary Table 8; Holliday et al., 2008; Kayal et al., 2011; Pelgas et al., 2011). We also identified DEGs related to plant defense pathways other than the phenylpropanoid pathway, such as the terpenoid pathway (Figure 5B; Supplementary Tables 4, 8), possibly indicating signaling crosstalk between secondary metabolites production and stress response (Jacobo-Velázquez et al., 2015; Isah, 2019).

### Identification of Transcription Factors Potentially Involved in the Transcriptional Control of Flavonoids

We identified 40 differentially expressed TFs from families (i.e., MYB, bHLH, WD40, WRKY and AP2/EREBP; Supplementary Table 6) known as involved in spruce PCs pathway (e.g., Bomal et al., 2008; Bedon et al., 2010; Nemesio-Gorriz et al., 2017). This is consistent with the fact that in higher plants, several R2R3-MYB proteins are known to activate the early steps of flavonoid biosynthesis, whereas late biosynthetic genes are rather controlled by the MYB-bHLH-WD40 (MBW) complex (Xu et al., 2015; Ma et al., 2018). In addition, recent studies reported that the action of the MBW complex can be modified by WRKY TFs (Lloyd et al., 2017). In this study, we found two R2R3-MYBs (GQ03719_G10, white spruce homolog of PaMYB33; and GQ04002_F03, white spruce homolog of PaMYB31) showing higher expression in high taxifolin glucoside content individuals (Supplementary Table 6), and for which protein-protein interactions with bHLH members were shown to play a role in the regulation of the flavonoid pathway in Norway spruce (Nemesio-Gorriz et al., 2017). Interestingly, the overexpression of PaMYB33 in transgenic cell lines also activated the expression of genes encoding PAL, ANR and LAR enzymes (Nemesio-Gorriz et al., 2017), suggesting that similar mechanisms may govern the late flavonoid biosynthesis in white spruce. The 12 TFs displaying opposite expression patterns between gallocatechin and taxifolin glucoside in our study (Supplementary Table 6) might be directly involved in the formation of the MBW complex or might be downstream target genes of the MBW complex. Given that the regulatory mechanism involving the MBW complex appears highly conserved in higher plants (Xu et al., 2015), our findings provide a relevant framework for studying the complex transcriptional network governing the biosynthesis of flavonoids, in white spruce and other conifer species.

### Integrating QTL Mapping and Transcriptomic Approaches: Potential, Limits and Perspectives

While RNA-Seq provided valuable insights into genes involved in white spruce phenylpropanoid pathway and their regulation, the combination of both QTL mapping and RNA-Seq approaches yielded mixed results. We identified 19 candidate DEGs colocalizing within QTLs, but none of them were formally associated with the PC pathway (Supplementary Table 7). However, considering that the landscape of genes essential for the regulation of phenylpropanoids in plants is still not exhaustive (for a review see Biala and Jasiński, 2018), we should not exclude the possibility that these candidate genes could be indirectly linked to the phenylpropanoid biosynthesis pathway. Further, the fact that one third (203) of the 603 DEGs identified herein have not been positioned yet on the current white spruce genetic map (Pavy et al., 2017) suggests that some additional DEGs could have colocalized with the detected QTLs. Association studies focusing on these DEGs would also help identify more candidate genes. Transcriptomics alone proved powerful to investigate the combinatorial gene regulation of flavonoids in white spruce. It allowed the identification of 137 genes likely co-regulated, along with several candidate regulators, and 50 genes encoding key enzymes of the white spruce phenylpropanoid pathway. These results may be further explored by focusing on the discovery of variable genomic regions responsible for variations in gene expression (i.e., eQTL studies), given that PCs regulatory networks appear relatively conserved in plants (e.g., MBW complex; see Xu et al., 2015).

## Data Availability Statement

The original contributions presented in the study are publicly available. This data can be found here: Concentrations in phenolic compounds (file: Concentration_metabolites.xlsx) were deposited in Dryad and are available at the following link: https://doi.org/10.5061/dryad.z08kprrcd. The raw read sequencing data was deposited in the European Nucleotide Archive (Accessions: ERR5545518, ERR5545555, ERR5545556, ERR5545558, ERR5545562, ERR5545566, ERR5545567, ERR5545569, ERR5545571, ERR5545573, ERR5545575, ERR5545578-ERR5545580, ERR5545583, ERR5545584, ERR5545586, ERR5545587, ERR5545591, ERR5545621-ERR5545624, ERR5545643-ERR5545645, ERR5545647-ERR5545649, ERR5545651-ERR5545653, ERR5545656, ERR5545657, ERR5545660, ERR5545661-ERR5545664, ERR5545666-ERR5545671, ERR5545673, ERR5545674, ERR5545676-ERR5545680, ERR5545682, ERR5545684-ERR5545687, ERR5545689-ERR5545695, ERR5545697-ERR5545699, ERR5545739-ERR5545743, ERR5545745, ERR5545746, ERR5545748-ERR5545753). The reference transcriptome (file: GCAT_WS-3.3.cluseq.fasta) used to map RNA-Seq reads was deposited in Dryad and is available at the following link: https://doi.org/10.5061/dryad.z08kprrcd.

## Author Contributions

JLao, AH, NI, and JB designed the study. JLao, SG, AH, AA, M-CG-L, BB, and JLar designed methods and carried out the experiments. JLao, CD, SG, CB, and ML performed the analyses and discussed the results. JLao and CD wrote the manuscript with contributions and feedbacks from SG, ML, CB, AA, M-CG-L, JLar, BB, AH, NI, and JB. All authors contributed to the article and approved the submitted version.

## Conflict of Interest

The authors declare that the research was conducted in the absence of any commercial or financial relationships that could be construed as a potential conflict of interest.

## Abbreviations

DEG: differentially expressed gene
DW: dry weight
Log_2_fc: log_2_ fold change
PCs: phenolic compounds
PVE: phenotypic variance explained
QTL: quantitative trait loci
sp. (spp.): Species.
