# Supplementary material for: Combining QTL Mapping and Transcriptomics to Decipher the Genetic Architecture of Phenolic Compounds Metabolism in the Conifer White Spruce
Source: Front Plant Sci. 2021 May 17;12:675108. doi: 10.3389/fpls.2021.675108 (PMC8166253; doi:10.3389/fpls.2021.675108)
Supplement: Supplementary file 1 [file Data_Sheet_1.pdf]

Supplementary Material

1 Supplementary Figures and Tables

1.1 Supplementary Figures

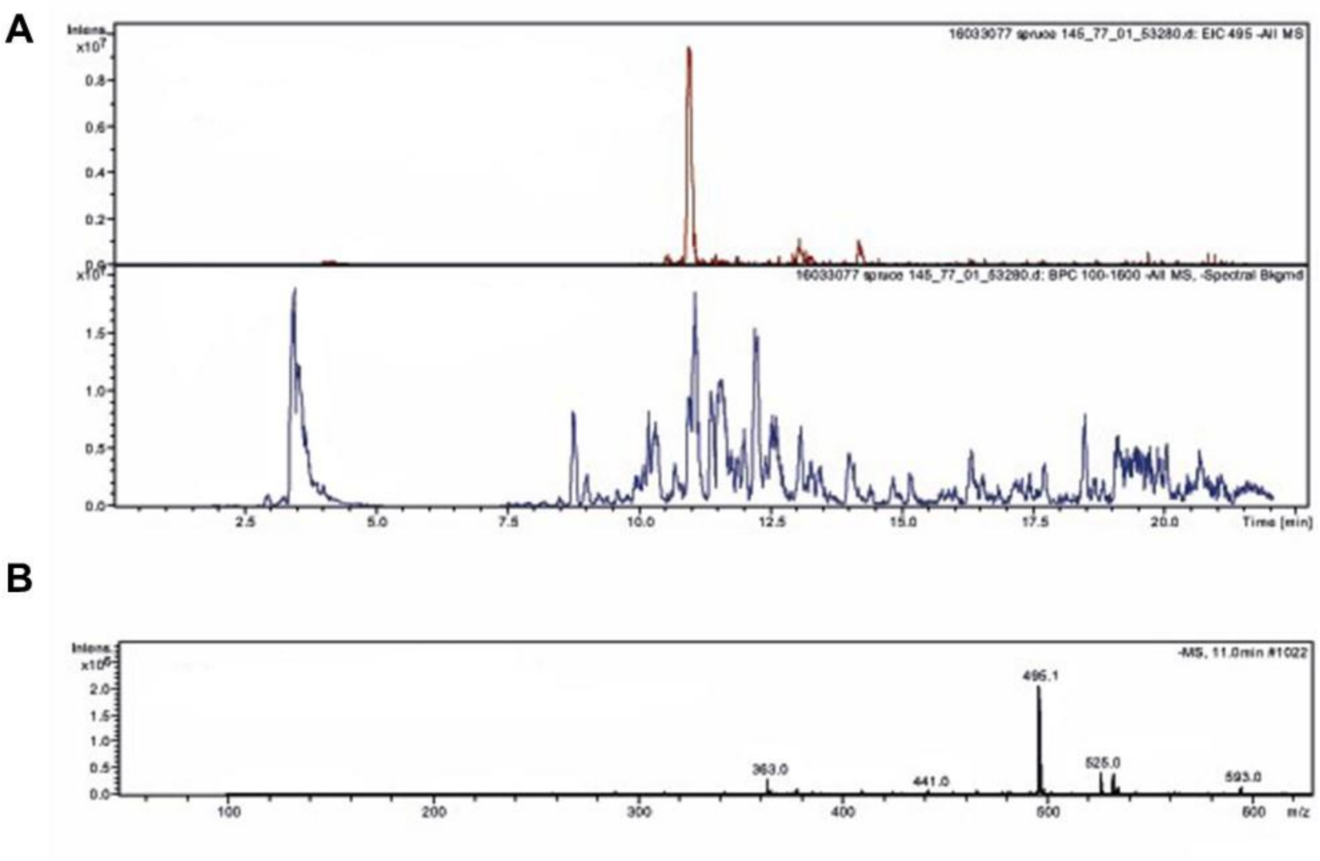

Supplementary Figure S1. Metabolic profile of *Picea glauca* and extracted ion chromatogram of neolignan-2. **(A)** Extracted ion chromatograms of neolignan-2 (upper part) and base peak chromatogram of all the molecular masses from 100-1600 Da detected in *P. glauca* upon chromatographic separation and detection in negative ionization mode (lower part). **(B)** Mass spectrum of neolignan-2. The molecular mass - *1* (MZ^{-1}) of 495.1 for neolignan-2 was detected with an in-source fragment of MZ^{-1}361 DA, representing the aglycone. Peak intensity is an arbitrary mass spectral unit denoting the relative ion content detected.

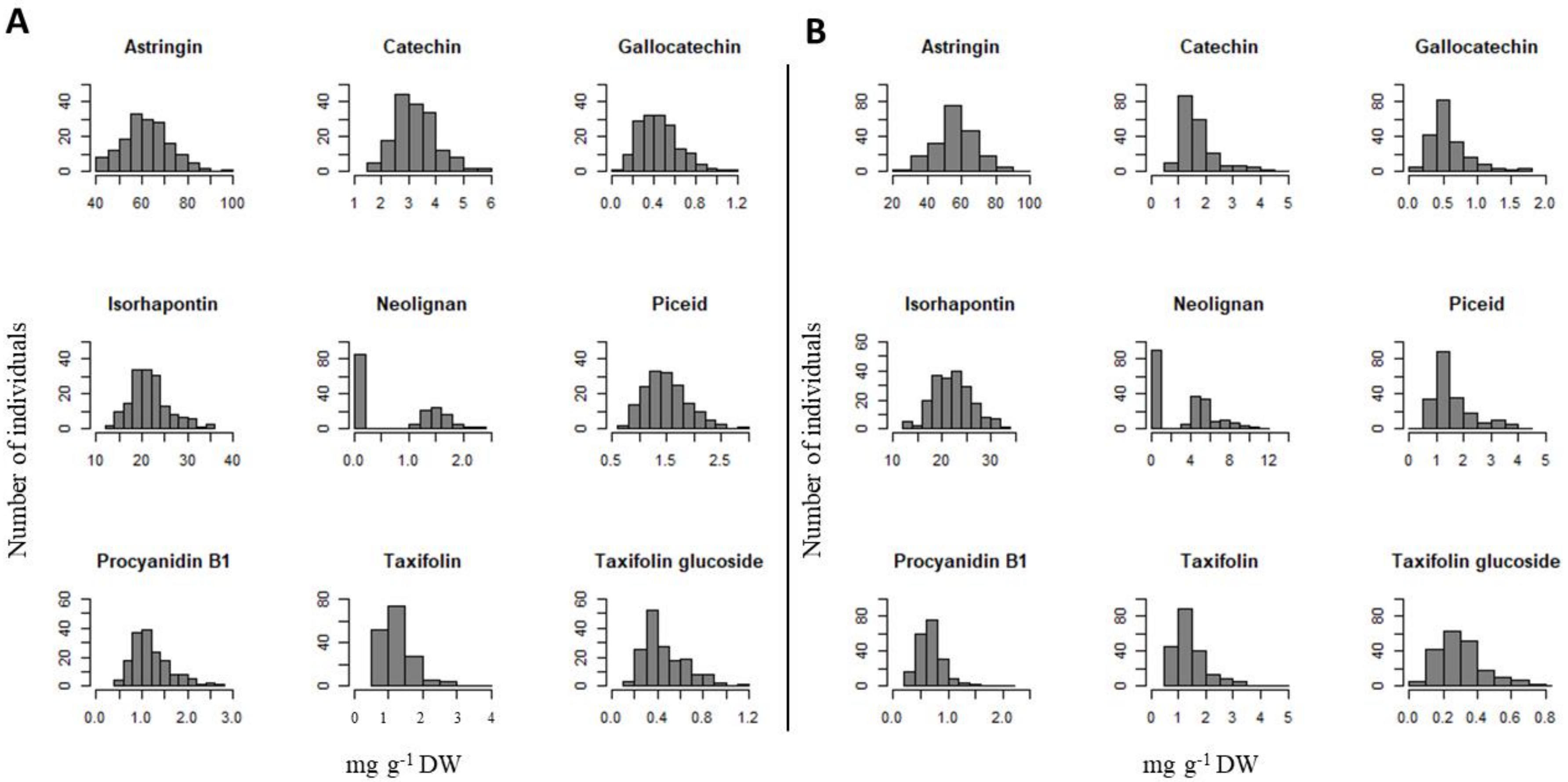

Supplementary Figure S2. Distributions of metabolite concentrations for nine phenolic compounds measured in 2014 **(A)** and 2017 **(B)** in a white spruce full-sib family (respectively 164 and 202 siblings from cross ♀77111 × ♂2388). DW: dry weight.

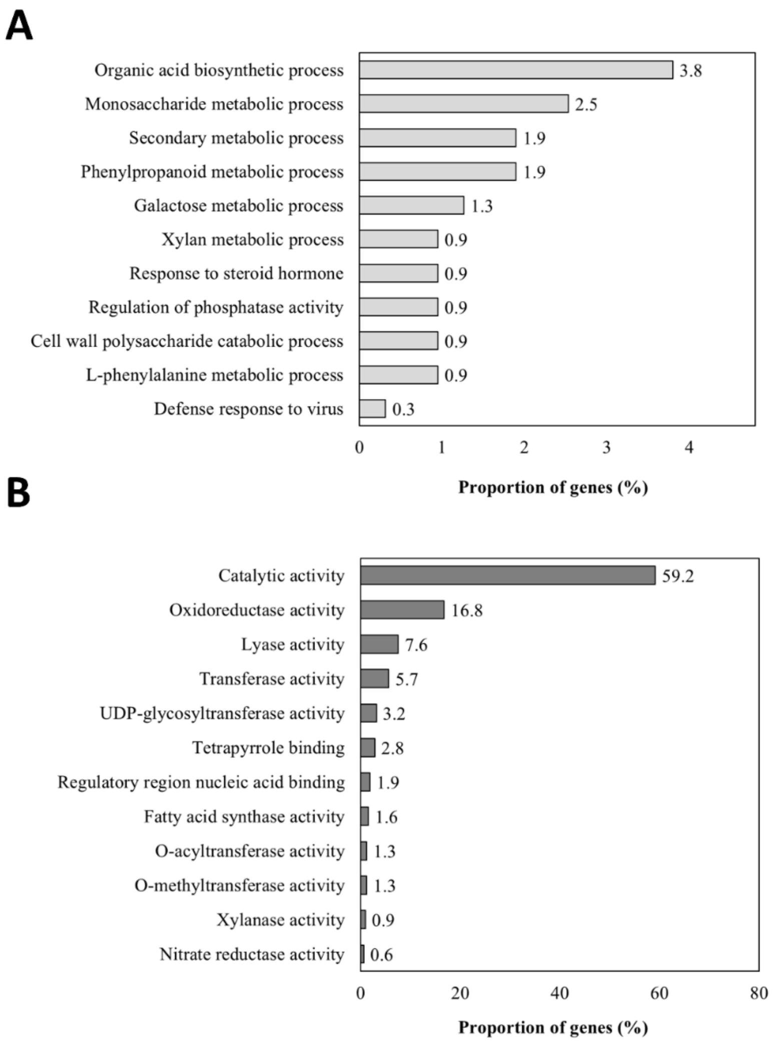

Supplementary Figure S3. GO enrichment analyses on differentially expressed genes (DEGs) identified for all five phenolic compounds (gallocatechin, neolignan-2, piceid, procyanidin B1 and taxifolin glucoside). Enrichment analyses were performed with Blast2GO using Fisher's exact tests ($P < 0.05$) for **(A)** biological processes and **(B)** molecular functions. Relevant functional categories are presented.

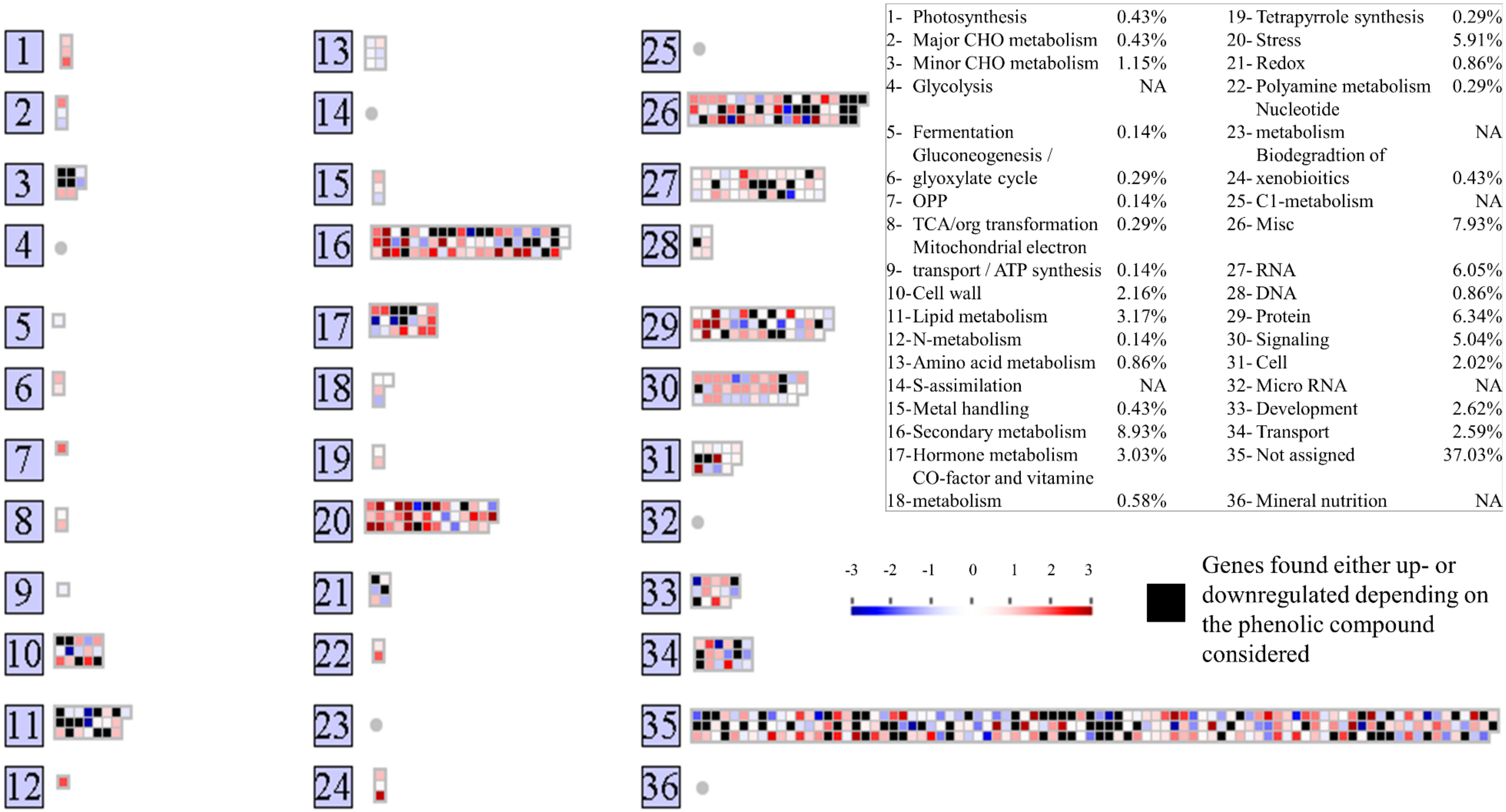

1- Photosynthesis	0.43%	19- Tetrapyrrole synthesis	0.29%
2- Major CHO metabolism	0.43%	20- Stress	5.91%
3- Minor CHO metabolism	1.15%	21- Redox	0.86%
4- Glycolysis	NA	22- Polyamine metabolism	0.29%
5- Fermentation	0.14%	23- Nucleotide metabolism	NA
6- Gluconeogenesis / glyoxylate cycle	0.29%	24- Biodegradtion of xenobioitics	0.43%
7- OPP	0.14%	25- C1-metabolism	NA
8- TCA/org transformation	0.29%	26- Misc	7.93%
9- Mitochondrial electron transport / ATP synthesis	0.14%	27- RNA	6.05%
10-Cell wall	2.16%	28- DNA	0.86%
11-Lipid metabolism	3.17%	29- Protein	6.34%
12-N-metabolism	0.14%	30- Signaling	5.04%
13-Amino acid metabolism	0.86%	31- Cell	2.02%
14-S-assimilation	NA	32- Micro RNA	NA
15-Metal handling	0.43%	33- Development	2.62%
16-Secondary metabolism	8.93%	34- Transport	2.59%
17-Hormone metabolism	3.03%	35- Not assigned	37.03%
18-CO-factor and vitamine metabolism	0.58%	36- Mineral nutrition	NA

Supplementary Figure S4. MapMan classification of genes differentially expressed (DEGs) among groups of progeny displaying contrasting phenotypes for five phenolic compounds (gallocatechin, neolignan-2, piceid, procyanidin B1 and taxifolin glucoside). DEGs were classified into different functional categories or 'BINs' (according to MapMan nomenclature). The percentage of DEGs assigned to each functional category is shown in the right frame.

		FLAVONOID					LIGNAN	STILBENE		
**Phenolic compounds**		Catechin	Gallocatechin	Procyanidin B1	Taxifolin	Taxifolin glucoside	Neolignan-2	Astringin	Isorhapontin	Piceid
**FLAVONOID**	Catechin		0.81***	0.83***	0.72***	0.20	0.32	0.81***	0.37	0.88***
	Gallocatechin	0.35		0.63**	0.57**	-0.17	0.29	0.59**	0.29	0.77***
	Procyanidin B1	0.86***	0.44**		0.59**	0.52*	0.09	0.57**	0.19	0.68***
	Taxifolin	0.78***	0.18	0.64**		0.39	0.31	0.58**	0.33	0.62**
	Taxifolin glucoside	0.79***	0.03	0.56**	0.88***		-0.10	0.12	-0.07	0.11
**LIGNAN**	Neolignan-2	0.36	-0.11	0.21	0.35	0.28		0.26	-0.10	0.40
**STILBENE**	Astringin	0.74***	0.29	0.55*	0.59**	0.53*	0.38		0.72***	0.79***
	Isorhapontin	0.64***	0.13	0.58**	0.63**	0.43	0.41	0.79***		0.34
		0.84***	0.32	0.71***	0.70***	0.65**	0.39	0.88***	0.73***	

Supplementary Figure S5. Pairwise phenotypic correlations between metabolite contents measured for taxifolin glucoside (bottom left) and gallocatechin (top right) extreme individuals examined in RNA-seq. Metabolites are classified into three major classes. The scale bar reports positive (red) and negative (blue) correlations. For each phenotypic correlation, the correlation coefficient and significance level are indicated. Levels of significance are as follows: * for $P < 0.05$, ** for $P < 0.01$ and *** for $P < 0.001$.

1.2 Supplementary Tables

Supplementary Table S1. Descriptive statistics of phenolic compounds measurements over two years of sampling in progeny trees of the white spruce ♀77111 × ♂2388 full-sib family.

Trait	Sampling year		Mean ± SD (mg g^{-1} DW)[a]	Min-Max[b]
Astringin	2014		62.35 ± 10.02	42.16-95.81
	2017		56.46 ± 12.26	23.33-92.79
Catechin	2014		3.26 ± 0.75	1.69-5.65
	2017		1.72 ± 0.72	0.61-4.56
Gallocatechin	2014		0.44 ± 0.20	0.07-1.17
	2017		0.59 ± 0.31	0.10-1.80
Isorhapontin	2014		21.65 ± 4.34	13.46-35.10
	2017		22.20 ± 3.94	12.08-32.30
Neolignan-2[c]	2014	High	1.56 ± 0.27	1.09-2.38
		Low	0.09 ± 0.02	0.05-0.15
	2017	High	5.82 ± 1.64	3.69-11.47
		Low	0.20 ± 0.13	0.06-0.75
Piceid	2014		1.49 ± 0.40	0.77-2.83
	2017		1.55 ± 0.74	0.48-4.05
Procyanidin B1	2014		1.20 ± 0.41	0.54-2.62
	2017		0.69 ± 0.27	0.24-2.03
Taxifolin	2014		1.25 ± 0.49	0.56-3.72
	2017		1.41 ± 0.54	0.60-3.16
Taxifolin glucoside	2014		0.46 ± 0.18	0.15-1.15
	2017		0.31 ± 0.14	0.05-0.79

[a]Mean and standard deviation (SD) of phenolic contents in the 164 and 202 siblings from cross ♀77111 × ♂2388 sampled in 2014 and 2017, respectively.
[b]Minimum and maximum phenolic contents observed among the samples trees.
[c]For neolignan-2, the mean was calculated on the two contrasted groups (high and low) of neolignan-2 content.

Supplementary Table S2. List and putative functions of genes identified in QTLs. The two candidate genes LAR (GQ03701_M12) and F3'5'H (GQ03712_G11) identified for procyanidin B1 and taxifolin glucoside QTLs, respectively are represented in bold. Those genes are known to be involved in phenolic compound biosynthesis in Norway spruce (Nemesio-Gorriz et al., 2016; Hammerbacher et al., 2018).

See separate excel file.

Supplementary Table S3. Summary of RNA-Seq and mapping statistics.

Sample ID	Number of reads in raw data	Number of reads following quality check	Number of reads mapped	Percentage of reads mapped (%)	Individuals with high metabolite concentrations[a]	Individuals with low metabolite concentrations[b]
PG_18	88,195,102	88,118,792	28,548,790	32.4		N, P, PB1, Tg
PG_21	84,180,862	83,864,050	27,577,315	32.9		N, Tg
PG_34	65,018,240	64,978,416	22,203,099	34.2	N	G, P
PG_44	80,972,822	80,944,648	28,379,002	35.1	N	
PG_49	81,150,742	81,058,788	26,403,407	32.6		N
PG_57	97,142,786	96,987,572	32,530,013	33.5		N, Tg
PG_59	70,066,502	70,028,654	23,862,501	34.1	N	Tg
PG_62	70,728,516	70,701,556	22,209,324	31.4		N, P
PG_73	40,479,338	40,435,368	13,631,424	33.7		N
PG_114	72,379,336	72,196,418	23,931,839	33.1		N
PG_131	97,689,388	97,244,160	31,287,585	32.2		N, P, PB1
PG_132	69,105,652	69,089,116	22,294,165	32.3	N	G, PB1
PG_163	62,132,100	62,036,128	20,560,167	33.1		N, P, PB1
PG_171	83,609,688	83,461,640	27,421,372	32.9		N
PG_227	82,057,992	81,928,752	27,489,476	33.6	Tg	
PG_232	80,305,232	80,248,044	27,951,720	34.8	G, N, P	
PG_235	81,080,968	81,013,378	26,567,423	32.8		N, PB1
PG_245	81,942,570	81,877,936	26,299,894	32.1	N, P	
PG_250	74,451,502	74,331,344	25,773,056	34.7	N, PB1	
PG_260	80,415,810	80,296,746	25,421,093	31.7	N	
PG_300	45,030,488	44,983,622	14,921,967	33.2	P	
PG_304	141,044,744	140,882,600	47,887,996	34.0	PB1	
PG_307	99,232,918	99,177,652	33,969,670	34.3	G, P	
PG_312	80,950,236	80,920,478	27,379,570	33.8		N
PG_320	76,581,338	76,528,942	26,181,750	34.2		N, Tg
PG_327	66,466,348	66,370,960	22,522,853	33.9	Tg	
PG_330	59,433,286	59,351,158	16,092,203	27.1	P, PB1	N
PG_331	75,375,134	75,347,566	25,781,473	34.2	Tg	N
PG_337	60,761,868	60,677,892	20,891,986	34.4	N, P, PB1, Tg	
PG_346	95,843,692	95,778,198	32,182,142	33.6	G, N, P	
PG_350	100,400,560	100,068,462	33,411,541	33.4	N	Tg
PG_377	92,035,488	91,761,854	29,692,432	32.4	Tg	
PG_385	72,070,622	71,720,028	24,237,796	33.8	N	
PG_390	89,686,948	89,584,342	30,634,917	34.2	G	N
PG_392	78,814,654	78,733,128	24,521,032	31.1	G, P, PB1, Tg	
PG_405	116,784,662	116,471,370	38,993,060	33.5	N, P	
PG_406	48,082,180	48,056,084	16,994,233	35.4	N	P, PB1
PG_409	132,941,612	132,847,158	45,040,807	33.9		N, PB1, Tg
PG_410	76,503,556	76,446,458	25,886,211	33.9	G, N, PB1	
PG_412	73,961,606	73,914,136	25,468,135	34.5	N	
PG_420	135,814,210	135,751,562	46,665,975	34.4	G	
PG_434	54,793,086	54,746,568	18,025,740	32.9	N	G
PG_448	78,519,580	78,500,014	25,579,503	32.6	G	N, Tg
PG_458	81,262,756	81,143,490	26,958,704	33.2		N
PG_470	109,720,664	109,378,552	34,048,256	31.1	N	PB1
PG_479	103,162,746	103,033,442	34,431,691	33.4		N
PG_492	77,288,962	77,239,724	24,661,160	31.9		G, N
PG_493	44,853,304	44,822,986	15,105,557	33.7	N	
PG_495	115,112,658	114,773,992	31,258,996	27.2		N, P, PB1
PG_527	136,403,518	136,348,178	46,696,040	34.2		G

Sample ID	Number of reads in raw data	Number of reads following quality check	Number of reads mapped	Percentage of reads mapped (%)	Individuals with high metabolite concentrations[a]	Individuals with low metabolite concentrations[b]
PG_533	133,986,932	133,346,208	42,722,116	32.0	N	G
PG_552	79,078,672	79,055,774	24,994,790	31.6	N	
PG_571	57,847,104	57,831,694	19,590,774	33.9	N	PB1
PG_579	65,691,352	65,671,112	21,418,734	32.6	N	
PG_593	81,795,664	81,692,504	27,758,140	34.0		N, Tg
PG_618	74,856,238	74,810,762	25,003,321	33.4	N	G
PG_630	72,850,398	72,821,466	24,070,741	33.1	N	
PG_658	70,938,262	70,910,244	23,804,444	33.6		N, P
PG_674	58,779,084	58,755,606	19,573,975	33.3	Tg	N
PG_677	35,660,038	35,539,676	12,162,598	34.2		N
PG_700	101,057,628	100,766,572	33,089,667	32.8	G, N	
PG_707	72,868,546	72,809,606	23,536,193	32.3	N	
PG_716	68,821,540	68,680,736	22,442,049	32.7		N
PG_733	75,430,996	75,376,194	25,523,375	33.9	PB1, Tg	
PG_744	66,110,412	66,036,228	21,967,985	33.3	N, PB1	
PG_747	80,299,962	80,275,222	26,642,124	33.2	Tg	N
PG_749	66,414,044	66,296,716	20,878,495	31.5	N, Tg	
PG_753	60,572,850	60,534,268	19,905,456	32.9	N	
PG_763	112,380,518	112,112,628	33,872,892	30.2	N, PB1	
PG_766	80,354,014	80,324,500	27,286,133	34.0		N, P
PG_787	76,813,878	76,677,700	23,933,264	31.2		N, P
PG_807	133,368,038	133,155,102	44,894,519	33.7	N	G
PG_829	56,885,504	56,855,736	19,533,620	34.4	N	
PG_848	99,989,104	99,571,970	32,590,057	32.7		G, N
PG_849	75,968,380	75,918,288	25,632,516	33.8	G, N, P	
PG_850	80,138,258	79,787,104	27,426,333	34.4	PB1	N
PG_856	163,017,710	162,821,342	53,971,250	33.1	N	
PG_905	65,034,536	65,011,958	21,820,589	33.6		N, Tg
PG_910	92,552,118	92,507,610	30,082,856	32.5		N
PG_916	77,432,032	77,339,570	26,007,740	33.6		G, N

[a,b] metabolites are named according to the following abbreviations: G=gallocatechin, N=neolignan-2, P=piceid, PB1=procyanidin B1 and Tg=taxifolin glucoside.

Supplementary Table S4. List of the 603 differentially expressed genes (DEGs) identified for each metabolite.
See separate excel file.

Supplementary Table S5. List of differentially expressed genes (DEGs) classified into BIN functions in MapMan.
See separate excel file.

Supplementary Table S6. Differentially expressed transcription factors (TFs) identified for all five phenolic compounds showing significant QTLs for the two years of measurement.
See separate excel file.

Supplementary Table S7. Differentially expressed genes (DEGs) located in QTLs. Sequence description: annotations obtained using BLAST2GO ($P < 0.05$); InterPro classification: most informative InterPro names.

Metabolite measured for QTL detection	Linkage group	Parents	Position on the consensus map	PVE	DEG ID	Sequence description	Interpro classification	Metabolite for which genes are differentially expressed	Adjusted p-value	Log_2fc	Expression
Gallocatechin	8	77111	93.8	6.6	GQ04112_C19	expansin-like	Expansin cellulose-binding-like domain	Gallocatechin	1.01E-02	0.99	-
			91.3	7.1	GQ03107_N12	uncharacterized protein ycf45	FY-rich C-terminal	Gallocatechin	1.35E-02	0.49	-
			86.9		GQ03208_O15	uric acid degradation bifunctional protein TTL	Transthyretin/hydroxyisourate hydrolase domain	Gallocatechin	3.46E-02	0.27	-
			107.3	6.6	GQ03006_J10	chaperone protein dnaJ 16	Protein trichome birefringence-like 12	Gallocatechin	2.30E-02	0.23	-
			97.5	6.1	GQ03108_K22	uncharacterized protein	Mur ligase C-terminal domain superfamily	Gallocatechin	4.74E-02	-0.26	-
			86.9		GQ03326_I17	protein ABIL1	TFB5-like superfamily	Gallocatechin	7.05E-03	-0.35	-
			97.6		GQ04004_J08	polyadenylate-binding protein-interacting protein 11-like	RNA-binding domain superfamily	Gallocatechin	4.74E-02	-0.38	-
			91.3	7.1	GQ03013_K19	uncharacterized protein	Acyl transferase	Gallocatechin	4.01E-03	-0.53	
Neolignan-2	4	2388	130.6		GQ0073_O14	salicylic acid-binding protein 2-like isoform X1	Alpha/beta hydrolase	Neolignan	2.44E-04	1.27	HIGH
			131.5		GQ03224_O05	endo-1,3;1,4-beta-D-glucanase-like isoform X2	Dienelactone hydrolase	Neolignan	6.92E-03	1.12	HIGH
			132.8		GQ04002_K24	WAT1-related protein At1g21890	EamA domain	Neolignan	3.15E-03	-1.01	LOW
			130.5		GQ03001_B05	methylesterase 3-like	Alpha/Beta hydrolase fold	Neolignan	1.45E-14	-2.46	LOW
			123.9		GQ03232_O21	tRNA(Ile)-lysidine synthase, chloroplastic-like	tRNA(Ile)-lysidine/2-thiocytidine synthase N-terminal	Neolignan	6.05E-05	0.33	-
			122.8	60.9	GQ03601_O07	fumarate hydratase 1, mitochondrial	Fumarate lyase family	Neolignan	1.03E-03	0.28	-
			130.6		GQ0173_N14	probable pyridoxal 5'-phosphate synthase subunit PDX1	Pyridoxal 5'-phosphate synthase	Neolignan	1.20E-02	-0.22	-
			129.6		GQ02820_P07	anthranilate N-benzoyltransferase protein 1	Chloramphenicol acetyltransferase-like domain superfamily	Neolignan	6.28E-12	-0.27	-
			122.8	61.1	GQ03302_G05	probable methyltransferase PMT11	Cupredoxin	Neolignan	2.60E-06	-0.32	-
			130.2		GQ04102_G23	methylesterase 3-like	Alpha/Beta hydrolase fold	Neolignan	2.23E-03	-0.79	-
Taxifolin glucoside	9	77111	2.1		GQ03322_E04	uncharacterized protein LOC21386978	Ubiquitin-like domain superfamily	Taxifolin glucoside	8.68E-03	0.51	-

Piceid and procyanidin B1 do not appear in this table, as no DEGs were found within QTL regions for these metabolites. DEGs showing higher or lower expression are referred to as HIGH and LOW in the Expression column, respectively.
PVE: phenotypic variance explained, expressed in percentage (%) for DEGs found on parental maps.

Supplementary Table 8. Candidate genes for adaptation to climate in other studies.
See separate file.

1.3 Supplementary Methods

Supplementary Methods S1. Extraction of phenolic compounds.

Samples were finely ground in liquid nitrogen using a mortar and pestle, then lyophilized at 0.34 mbar pressure using an Alpha 1-4 LD plus freeze dryer (Martin Christ GmbH, Osterode, Germany). 20 mg of tissue powder was extracted twice for 4 h with 800 µl analytical grade methanol containing 10 µg ml^{-1} of apigenin-7-glucoside, an internal standard (Carl Roth GmbH, Karlsruhe, Germany). Phenolics were analyzed by LC-tandem mass spectrometry on an Agilent 1200 HPLC system (Agilent, Santa Clara, CA, U.S.A.) coupled to an API 3200 mass analyzer (Sciex, Darmstadt, Germany)as follows: *m/z* 303.0 → 125.0 (CE, -28 V; DP, -40 V) for taxifolin; *m/z* 465.0 → 285.0 (CE, -30 V; DP, -70 V) for taxifolin glucoside; $m\ z^{-1}$ 299.9 →109.1 (CE -34 V; DP -30 V) for catechin; $m\ z^{-1}$ 304.8 →179 (CE -28 V; DP -390 V) for gallocatechin; $m\ z^{-1}$ 576.9 →289.1 (CE -30 V; DP -50 V) for proanthocyanidin B1; $m\ z^{-1}$ 404.9 →243 (CE -31 V; DP -52 V) for astringin; $m\ z^{-1}$ 418.9 →257.1 (CE -30 V; DP -52 V) for isorhapontin; $m\ z^{-1}$ 389 →227 (CE -34 V; DP -50 V) for piceid; and $m\ z^{-1}$ 495 →162 (CE -38 V; DP -70 V) for neolignan-2 ((*2S*,*3S*)-2,3-dihydro-2-(4-hydroxy-3-methoxyphenyl)-3-(hydroxymethyl)-1,4-benzodioxin-6-propanol-xyloside). To develop the MRMs, standards for naringenin, eriodictyol, and taxifolin were purchased from Sigma-Aldrich, or from TransMIT GmbH Giessen, Germany for taxifolin-glucoside. Standards for the stilbenes were isolated from bark following the protocols described by Wadke et al. (2016). MRMs for compounds commercially unavailable were developed by using semi-crude plant extracts. Both Q1 and Q3 quadrupoles were maintained at unit resolution. Analyst 1.5 software (Applied Biosystems) was used for data acquisition and processing. Linearity of compound detection for quantification was verified by external calibration curves for catechin, taxifolin, astringin and proanthocyanidin B1. Phenolic compound concentrations were determined relative to the calibration curve of the compound most closely resembling it.

Supplementary Methods S2. RNA extraction.

Total RNA was isolated from frozen tissues following the method of Chang et al. (1993), with modifications listed in Pavy *et al.* (2008). Frozen bark samples (1 g) were ground to a very fine powder

using a MixerMill MM 400 (Retsch®, Haan, Germany) during 30s. The powder was transferred into a 1.5 ml Eppendorf® tube and 100 mg of the grounded tissue was used for the extraction using the CTAB protocol. RNA was extracted with 750 µl of extraction buffer (1 ml/sample cetyl trimethylammonium bromide (CTAB (1L): 20 g polyvinylpyrrolidone (PVP), 20 g CTAB, 100 mL Tris-HCl 1M pH 8.0, 50 mL ethylenediaminetetraacetic acid (EDTA) 0.5 M pH 8.0, 400 mL NaCl 5M, 0.5 g spermidine) and 2% β-mercaptoethanol that had been preheated to 65°C, after which samples were vigorously vortexed. Tissues were incubated at 65°C in a water bath for 10 min with vortexing periodically. A volume of 500 µl of chloroform:isoamyl alcohol (24 : 1 v/v) was added, and tubes were vortexed and centrifuged for 5 min at 15 000 rpm at 4°C in centrifuge 5417 R (Eppendorf®, Hamburg, Germany). The supernatant was transferred into a new tube and was reextracted with another 500 µl of chloroform:isoamyl alcohol. The combined aqueous phases were transferred into a new tube, an equal volume of LiCl-EDTA (7.5 M LiCl and 50 mM EDTA) was added, and RNA was precipitated at –20°C for 1 h. RNA was pelleted by centrifugation at 15 000 rpm g at 4°C. The pellet was rinsed with 500 µl of 80% ethanol, collected by 5 min of centrifugation at 15 000 rpm at 20°C. This step was done twice. After drying the pellet for 15 min in the open air, the pellet was re-suspended in 30 µL of autoclaved nanopure water. The quality of total RNA was assessed using the Agilent 2100 Bioanalyzer with RNA 6000 Nano LabChips (Agilent Technologies Inc, Santa Clara, CA, U.S.A.) and RNA concentration was determined with a Nanodrop 1000 spectrophotometer (Thermo Fisher Scientific, Wilmington, DE, U.S.A). For all samples, RNA concentration exceeded 100 ng/µl and RNA Integrity Number (RIN) exceeded 7.8.

References

Chang, S., Puryear, J., and Cairney, J. (1993). A simple and efficient method for isolating RNA from pine trees. *Plant Mol. Biol. Rep.* 11, 113–116. doi: 10.1007/BF02670468

Pavy, N., Pelgas, B., Beauseigle, S., Blais, S., Gagnon, F., Gosselin, I., et al. (2008). Enhancing genetic mapping of complex genomes through the design of highly-multiplexed SNP arrays: application to the large and unsequenced genomes of white spruce and black spruce. *BMC Genomics* 9, 21. doi: 10.1186/1471-2164-9-21

Wadke, N., Kandasamy, D., Vogel, H., Lah, L., Wingfield, B.D., Paetz, C., Wright, L.P., Gershenzon,, J., Hammerbacher, A. (2016). The bark-beetle-associated fungus, *Endoconidiophora polonica*, utilizes the phenolic defense compounds of its host as a carbon source. *Plant Physiol.* 171, 914–931. doi: 10.1104/pp.15.01916
